# Comparative genomics and functional analysis of the 936 group of lactococcal *Siphoviridae* phages

**DOI:** 10.1038/srep21345

**Published:** 2016-02-19

**Authors:** James Murphy, Francesca Bottacini, Jennifer Mahony, Philip Kelleher, Horst Neve, Aldert Zomer, Arjen Nauta, Douwe van Sinderen

**Affiliations:** 1 School of Microbiology, University College Cork, Cork, Ireland; 2 Alimentary Pharmabiotic Centre, University College Cork, Cork, Ireland; 3 Department of Microbiology and Biotechnology, Max Rubner-Institut, Kiel, Germany; 4 FrieslandCampina, Amersfoort, The Netherlands

## Abstract

Genome sequencing and comparative analysis of bacteriophage collections has greatly enhanced our understanding regarding their prevalence, phage-host interactions as well as the overall biodiversity of their genomes. This knowledge is very relevant to phages infecting *Lactococcus lactis*, since they constitute a significant risk factor for dairy fermentations. Of the eighty four lactococcal phage genomes currently available, fifty five belong to the so-called 936 group, the most prevalent of the ten currently recognized lactococcal phage groups. Here, we report the genetic characteristics of a new collection of 936 group phages. By combining these genomes to those sequenced previously we determined the core and variable elements of the 936 genome. Genomic variation occurs across the 936 phage genome, such as genetic elements that (i) lead to a +1 translational frameshift resulting in the formation of additional structures on the phage tail, (ii) specify a double neck passage structure, and (iii) encode packaging module-associated methylases. Hierarchical clustering of the gene complement of the 936 group phages and nucleotide alignments allowed grouping of the ninety 936 group phages into distinct clusters, which in general appear to correspond with their geographical origin.

Extensive research efforts have been dedicated to (bacterio)phages infecting *Lactococcus lactis* starter cultures, providing insights into phage-host interactions^1^ and generating detailed knowledge on structural aspects of the *Siphoviridae* phage particle^2^. Phages isolated from dairy fermentation facilities that utilise *L. lactis* as a primary starter culture predominantly represent members of the so-called 936 phage group^3^,^4^. These double-stranded (ds)DNA (26–32 kb) phages possess a long, non-contractile tail (130-165 nm) and an isometric capsid (45–60 nm), and exhibit a modular genetic architecture similar to other *Siphoviridae* phages comprising of late-, early- and middle-expressed gene modules^4^,^5^. Since the first 936 group phage, sk1, was sequenced^6^, the full genomes of an additional fifty four 936 group phages, with various geographical origins, have become available^3^,^4^,^7^,^8^. Most of the genes that correspond to the late transcript (representing the packaging, morphogenesis and cell lysis modules) are highly conserved among the individual genomes, despite their different geographical origins^9^. Although the greatest sequence diversity is found within the early and middle-expressed region (also referred to as the DNA replication module), other notable points of genetic divergence can be discerned within the morphogenesis (late-expressed) module, including differences among genes that encode the receptor binding protein (RBP), the tape measure protein (TMP) and the so-called neck passage structure (NPS)^3^,^10^,^11^. Despite these insights into the various genomic regions, the core and non-core genome, as well as the overall genomic diversity of all its sequenced members has yet to be fully characterised. Advances in genome sequencing technologies and bioinformatic tools have greatly aided in the understanding of phage genome diversity, reminiscent of developments seen for bacterial genomes^12^. Previous studies have demonstrated that the genomic content of tailed bacteriophages consists of core and accessory genes, the latter of which are believed to move horizontally among phages and to confer distinct properties^13^,^14^,^15^. In an effort to reveal the genetic make-up and genomic diversity of this dominant phage group at high resolution, we performed an analysis of 936 group phages at both the nucleotide and protein level using a Markov Cluster Algorithm (MCL). Firstly, we report on the complete genome sequences of thirty five novel 936 group phages isolated from four dairy plants. By combining a total of ninety different 936 group phages, a comparative protein analysis revealed the core and variable genome. Comparative analysis of the analysed phage genomes revealed various regions of diversity, which are likely to have arisen due to host-mediated phage resistance systems and/or man-made, processing-imposed hurdles to reduce their number and infectivity.

## Results

### The core genome of 936 group phages revealed by the Markov Cluster Algorithm

**Figure 1:**
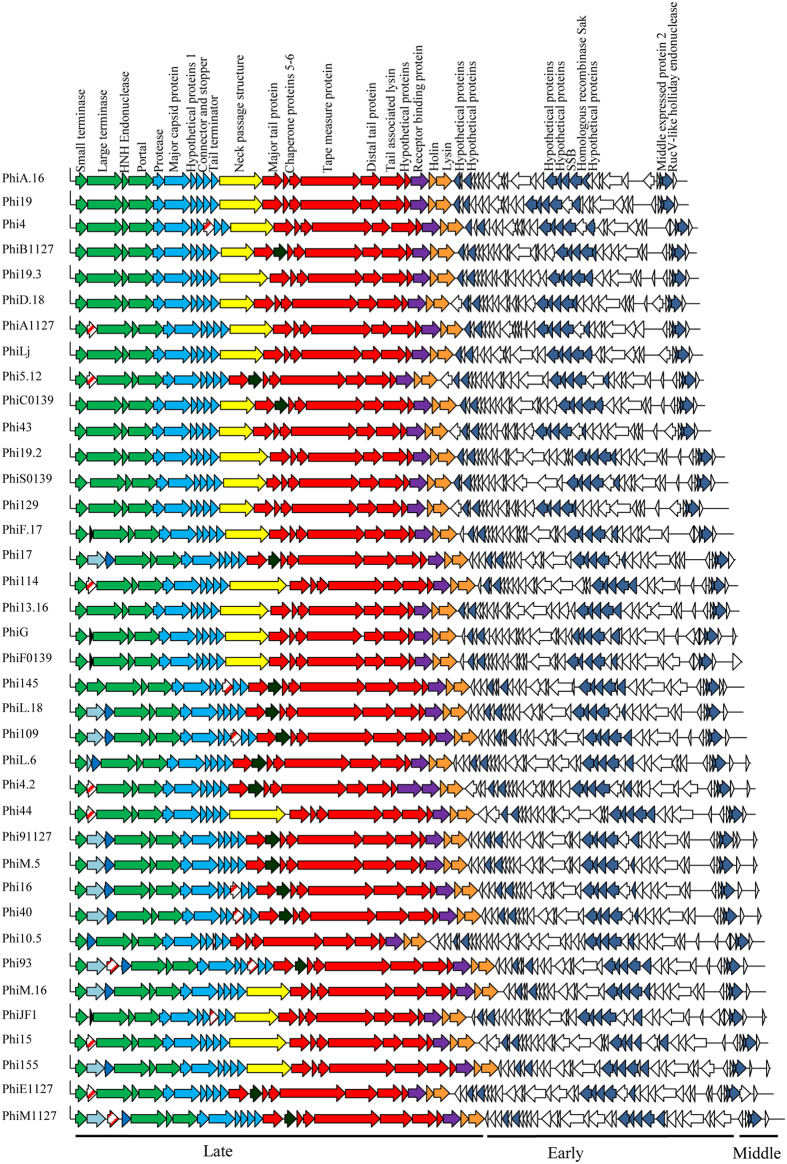
Schematic representation of the genetic architecture of the thirty eight phages sequenced in this study. Conserved ORFs are colour coded with green arrows representing the packaging module, blue arrows representing the capsid morphogenesis module, red arrows representing the tail morphogenesis module, purple arrows correspond to the RBP-encoding gene and orange arrows representing host cell lysis module, Blue/grey arrows represent a 5′-GGWG^m6^A-3′-specific methylase-encoding gene, Dark blue arrows encompass a 5′-G^m6^ATC-3′-specific methylase-encoding gene, Black and open (non-coloured) arrows represent genes with no known function, red striped arrows represent HNHE-encoding genes, yellow arrows represent genes encoding the NPS and dark green arrows represent *tpeX*. Dark blue arrows in the early and middle transcript represent the core genes located in the replication module.

**Figure 2:**
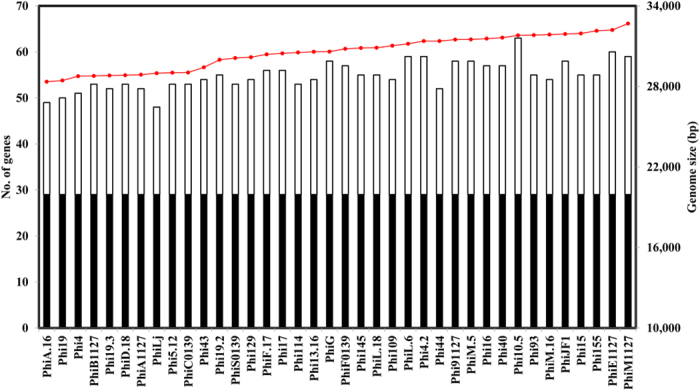
A graphical representation of the variability in non-core ORFs in the thirty eight 936 group phages. Black bars represent the core genome, white bars represent the non-core genes and the red line indicates genome size (bp).

To determine the core genome of the 936 group phages, thirty five phage genome sequences derived from a recently assembled collection, members of which had originated from four dairy factories, designated F1–F4, in The Netherlands (whose salient features are summarized in Table 1), were added to those sequenced previously, giving a total collection of ninety phages. The core genome of this phage group was determined by performing a BlastP all-against-all of the deduced amino acid sequences encoded by the gene pool extracted from each phage. The resulting BlastP outputs were then sorted using the Markov Cluster Algorithm (MCL) by grouping homologs, paralogs, orthologs and unique protein sequences (resulting in a total of one hundred and eighteen protein families; see Materials and Methods). A set of twenty nine protein families were identified to be commonly encoded by all examined phage genomes and thus believed to correspond to the core genome of the 936 group of lactococcal phages (i.e. the collection of protein families of which a member is found in every genome of all analysed 936 group phages)^16^. The genetic elements of the core genome primarily correspond to the conserved late transcript which encompasses twenty ORFs involved in the formation of the phage particle, DNA packaging, host cell recognition and lysis (Fig. 1). The other nine core ORFs are spread across the early and middle transcript and (are predicted to) encode five hypothetical proteins, a single-stranded DNA binding (SSB) protein, the homologous recombinase Sak (sensitivity to AbiK) protein, middle-expressed protein 2 and a RuvC-like Holliday endonuclease (Fig. 1). The core genes and their corresponding locus tags in the ninety phage genomes are listed in supplementary text file 1. The remaining eighty nine protein families represent the non-core genome which consists of non-conserved orthologs and variable/unique genes. In this study, the average number of variable genes in bacteriophages isolated from each factory was 24.92 ± 2.90 (*n* = 12), 23.75 ± 3.20 (*n* = 4), 27 ± 3.06 (*n* = 14), and 26.25 ± 3.30 (*n* = 8), for F1, F2, F3 and F4, respectively, with no correlation observed with genome size, year of isolation or the factory from which the phage originated (Fig. 2). This variability and apparent loss/gain of certain ORFs is also evident in previously sequenced genomes of representatives of this phage group (data not shown).

### Phage DNA packaging genes

In dsDNA phages the DNA packaging complex consists of the small and large terminase, TerS and TerL, respectively, the encoding gene of the latter often positioned immediately downstream of that of the former^17^. Comparison of the ninety genomes revealed that the majority (65.55%) harbour one or more ORFs between the predicted *terS* and *terL*. These interposing ORFs represent various predicted functions including a homing endonuclease (HNHE), a hypothetical protein and orphan methyltransferases (MTases) (Fig. 1). Homologs of these MTases in Phi93 and Phi145 have been shown to methylate phage DNA, providing a protective effect against host-encoded restriction endonucleases^7^.

### Genetic variation surrounding the major tail protein

**Figure 3:**
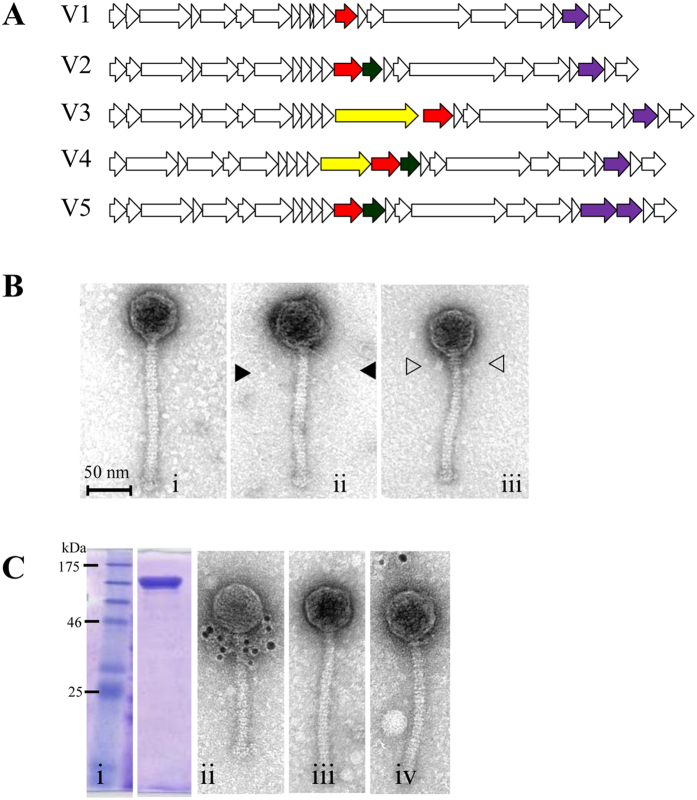
(**Panel A**) Schematic representation of the five different types of gene arrangements in the structural module of the sequenced phages. **V1:** no *nps* or *tpeX*; **V2:** *tpeX* downstream of *mtp*; **V3:** *nps* upstream of *mtp*; **V4:** *nps* gene upstream and *tpeX* downstream of *mtp*. **V5:** *tpeX* downstream of *mtp* and secondary *rbp* upstream of ‘standard’ *rbp*. Red arrow = *mtp*; Dark green arrow = *tpeX*; Yellow arrow = *nps*; Purple arrow = *rbp*. (**Panel B**) TEM of representative 936 group phages highlighting different morphological features: **i)** Phi16: no additional structures, **ii)** Phi4: single disc neck passage structure (NPS) with long thin whiskers and small terminal knots (▶), (**iii)** Phi15: double disc NPS with short whiskers and large terminal knots (▷) (morphotyping determined previously^36^). (**Panel C: i)** 12% SDS-PAGE of purified Phi15-NPS. **(ii)** Micrographs from immunogold TEM analysis of Phi15 labeled with polyclonal antibodies raised against Phi15-NPS confirming that the product of *npsPhi15* is located between the phage capsid and tail as predicted. Immunogold labelling was also performed on two phages as negative controls; (**iii)** Phi17 (without NPS structure), (i**v)** PhiLj (with single disc NPS and long whiskers).

The lactococcal 936 group phage p2 has been used as a model to elucidate the function of many of the conserved genes located within the late-transcribed module including those involved in phage particle morphogenesis^9^. In comparison to p2, it appears that the region surrounding the major tail protein-encoding gene (*mtp*) is a hotspot for gene rearrangements in several 936 group phages, including various members of our newly acquired phage collection (Fig. 3A). These include the presence/absence of a gene (designated *nps*) encoding a predicted Neck Passage Structure (NPS; a structure visible between the capsid and tail) upstream of the *mtp* and/or a gene encoding a tail extension protein (*tepX*; see below) downstream of the *mtp.* Morphologically, several features can be observed between the capsid and tail on EM images; no NPS (Fig. 3Bi), an NPS with long or thin whisker-like appendages ending in terminal knots (Fig. 3Bii) or a double disc NPS (Fig. 3Biii). Based on the genetic architecture, we assigned phages sequenced in this study to one of several groups, ranging from group V1 through to V5, based on the gene complement present in the *mtp* region (Table 1). These findings are reminiscent of those observed previously for certain 936 group phages, for which the genomic region around the MCP-encoding gene was shown to be a hotspot for genetic recombination^3^.

### Structural implications of the genetic variation surrounding the *mtp*

To assess the physical location of the protein products of *nps* and *tpeX*, a combination of electron microscopy, heterologous gene expression and Western hybridisation was employed. Using Phi15 as a representative of a 936 group phage possessing a double disc NPS (Fig. 3Biii), the location of the predicted NPS on the virion particle was confirmed using immunogold electron microscopy analysis. Antibodies that had been raised against recombinantly produced Phi15-NPS (Fig. 3Ci) were found to completely decorate the NPS-structure situated between the phage capsid and tail (Fig. 3Cii). No interaction was observed with Phi17, which lacks an NPS (Fig. 3 Ciii), or PhiLj, which harbours a single disc NPS (Fig. 3Civ). This indicates that certain phage isolates encode rather unique NPS structures.

**Figure 4:**
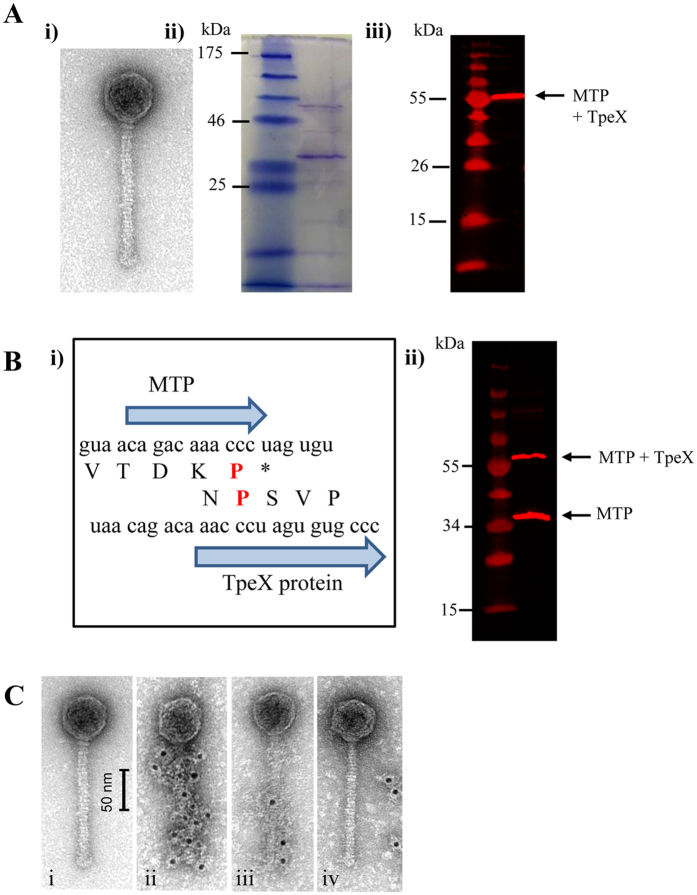
Characterisation of TpeX from PhiC0139. (**Panel A**) **i**) TEM of negatively stained phage PhiCO139 particle with unique thin spiral shaped tail fibers winding over the phage tail. **ii)** 12% SDS-PAGE of CsCl PhiC0139 viral particles. (**iii)** Western hybridisation analysis of CsCl PhiC0139 viral particles developed with polyclonal antibodies raised against TpeX. (**Panel B i)** Schematic representation of the nucleotide and amino acid sequence at the 3′ end of the *mtp* and 5′ end of the *tpeX* highlighting the potential +1 frameshift in red. (**ii)** Western hybridisation analysis of CsCl PhiC0139 viral particles developed with polyclonal antibodies raised against MTP from phage P2. (**Panel C i)** TEM of negatively stained phage PhiCO139 particle with unique thin spiral shaped tail fibers winding over the phage tail. (**ii)** Micrographs from immunogold TEM analysis of PhiC0139 labelled with polyclonal antibodies raised against purified TpeX confirming the spiral shapes of these tail decorations. Immunogold labeling was also performed on two phages to confirm that the PhiC0139 encodes a unique *tpeX*. (**iii)** phage P1082 (new isolate from MRI collection with similar spirals on tails). (**iv)** negative control with phage P008 (no spiral tail fiber).

Phages PhiC0139 and PhiB1127 (morphology group V4, Fig. 3A and data not shown) were found to possess an additional decorative structure in what appears to be a spiral-like shape extending across the length of the tail not observed in other 936 group phages (Fig. 4Ai). Using PhiC0139 as a representative, the *tpeX* gene, homologs of which are present in twenty one of the ninety 936 phages, and which is located immediately downstream of the *mtp*, was analysed further. TpeX-specific polyclonal antibodies were found to react strongly with a single protein of PhiC0139 viral particles separated by SDS-PAGE (Fig. 4Aii), although the molecular weight of this protein was significantly larger (~56 kDa) than that expected of TpeX (Fig. 4Aiii). This molecular weight would be consistent with the combined molecular weight of MTP (32.757 kDa) and TpeX (22.6 kDa). Sequence inspection of the 3′-end of *mtp* and the 5′-start of *tpeX* revealed the potential for a programmed +1 translational frameshift (Fig. 4Bi), which has previously been observed in the structural modules of various bacteriophages^18^,^19^. The presence of such a ‘shifty stop’ in the sequence CCC.UAG would lead to continued translation and thus fusion of MTP to TpeX (Fig. 4Bi). Western hybridisation analysis of SDS-PAGE-separated PhiC0139 viral particles employing antibodies against the MTP of phage p2 (the MTPs from p2 and PhiC0139 have 86% amino acid identity)^9^ revealed that the antibodies reacted with two PhiC0139 proteins, one with a molecular weight consistent with MTP and a second protein with a molecular weight that corresponds to the MTP-TpeX fusion (Fig. 4Bii). The (gold particle-labelled) antibodies raised against TpeX were furthermore found to decorate the phage tail of PhiC0139 in a ‘spiral’ like formation (Fig. 4Cii), unlike the uniform association of anti-MTP to the tail of 936 group phage p2^20^. A weak interaction occurred with the 936 group phage P1084 (Fig. 4Ciii), which was also found to have a spiral-like decoration (data from the Max Rubner-Institut collection), while no interaction occurred with phage P008 (Fig. 4Civ) which lacks spiral-like decorations. These findings indicate that the tail of PhiC0139 is composed of a mixture of MTP and MTP-TpeX subunits, the latter formed by programmed frame shifting. Furthermore, the MTP-TpeX subunits seem to be incorporated into the tail in an organised manner so as to form the visible spiral structures unique to the tail of PhiC0139 (Fig. 4C), this unique feature possibly being caused by particular amino acid differences between TpeX of PhiC0139 and equivalents encoded by other phages (Fig. S1). Interestingly, HHPRED analysis of TpeX indicates the presence of a carbohydrate-binding domain (*Cellulomonas fimi* E-value = 9.4e−06), thus suggesting that TpeX plays a role in phage-host interaction.

### Host cell recognition

**Figure 5:**
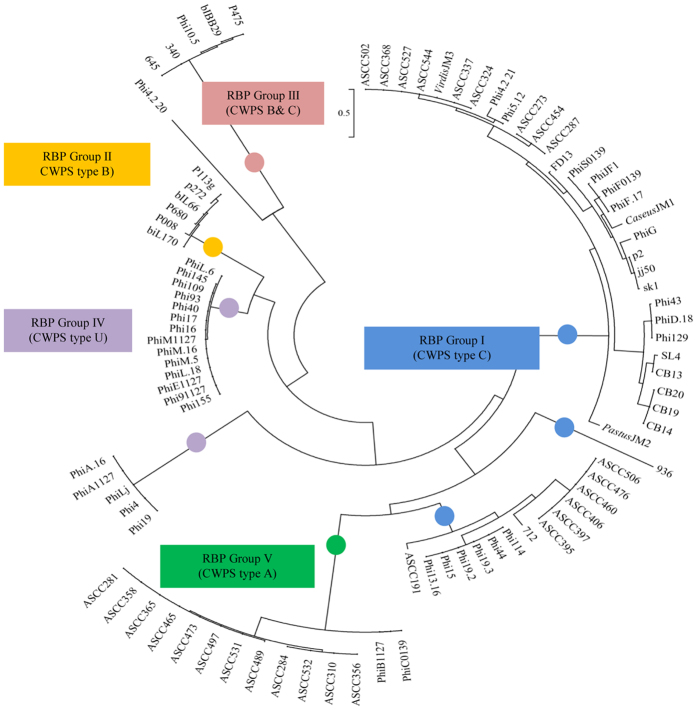
Phylogenetic tree of the C-terminal region of the RBP from all sequenced isolates from the 936 group of phages. The RBPs from the 936 group phages are grouped based on the known CWPS of the host strains. CWPS type C/RBP group I is marked blue; CWPS type B/RBP group II is marked yellow, CWPS type B&C/RBP group III is marked maroon, CWPS type U/RBP group IV is marked purple and CWPS type A/RBP group V is marked green.

Attachment of the 936 group phages to the surface of the target cell is mediated by the interaction of the phage receptor binding protein (RBP) and the bacterial cell wall polysaccharide (CWPS). Based on the genetic composition of the gene cluster responsible for CWPS biosynthesis, *L. lactis* strains have been divided into three types, designated type A, B and C (where type C can be further subdivided into five subtypes, designated C1 through to C5). It is believed that each lactococcal (sub)type produces a CWPS of a particular saccharidic structure which acts as the surface receptor for the 936 group, P335 group and so-called rare lactococcal 949, P087 and 1358 phage groups^1^,^8^,^21^,^22^. Comparative sequence analysis of the C-terminal region of the RBPs of 936 group phages revealed three RBP groups (I–III), which correlate to the CWPS type of the corresponding host(s) of such phages^8^. As part of the current study, the CWPS gene clusters of the host strains were typed and correlated to this RBP phylogeny. Sixteen of the thirty eight newly sequenced phages fall within the previously identified group I (which corresponds to hosts that possess a CWPS type C gene cluster), while one falls within group III (corresponding to CWPS types B & C; Fig. 5). Several RBPs appear to form a new group infecting strains with an unknown CWPS type (Group IV). The two RBPs encoded by phages PhiC0139 and PhiB1127 form a separate RBP group together with RBPs from phages that had been characterized in an Australian study^3^. This RBP group was designated here as group V (Fig. 5) and represent 936 group phages that are capable of infecting a lactococcal host with a type A CWPS gene cluster. Notably, the Phi4.2 genome encompasses two adjacent genes each predicted to encode a RBP protein (corresponding to locus tags Phi4.2_20 and Phi4.2_21) with the RBP encoded by Phi4.2_*orf21*_ aligning with group I, while the RBP encoded by Phi4.2_*orf20*_ appears to form its own subgroup, perhaps interacting with an as yet undefined CWPS (Fig. 5). The deduced Phi4.2_*orf20*_ product (376 aa) is considerably larger than previously sequenced 936-RBPs (which range in length from 253 to 268 aa) sharing a maximum percentage amino acid identity of 55% with the RBP of 936 group phage Q62 (Genbank: AAT81514).

### The early and middle transcripts: A genetic enigma of gene gain and loss

The DNA region corresponding to the early and middle transcripts (also designated as the replication region) exhibits the highest level of sequence diversity among genomes of 936 group phages. With a few exceptions, none of the identified ORFs have assignable functions due to the lack of significant sequence similarity to annotated sequences in public databases. As mentioned above, ORFs encompassed by the early transcript with a predicted or proven function include those that encode a putative DNA polymerase subunit, the SAK and SaV proteins and a SSB protein^23^,^24^,^25^. The genes that correspond to the middle transcript of the 936 group phages are believed to play a role in the processing of replication intermediates, such as Holliday junctions (HJs)^26^. Of the various replication-associated ORFs, only nine were determined to belong to the core genome and are spread across the early and middle transcript regions (Fig. 1). The replication region appears to be particularly prone to gene variability as evident by a difference of ten variable ORFs between the largest and smallest phage genome characterized in this study, i.e. those of PhiA.16 and PhiM1127, respectively. A small number of these non-core genes have been functionally characterised with regard to their role as targets in host-encoded abortive infection (Abi) systems and the presence of these targets varies across the sequenced phages. Phage 712 was found to lack the *sav* gene, while phage ASCC356 lacks the gene encoding middle expressed protein 1, both of which have been shown to acquire point mutations that negate the activity of AbiV and AbiD1, respectively^23^,^27^. Similarly, in phage P008, ORF41 is known to act in circumventing the activity of the two-protein anti-phage system AbiT, yet homologs of this gene are not present in seven 936 group phages. Finally, it has been demonstrated that the acquisition (via homologous recombination) of bIL170 phage gene *e6* (corresponding to locus tag bIL170p54) by phage bIL66M1 circumvents the activity of AbiP. Of the ninety phages used in this study, homologs of bIL170p54 were identified in just three phages, P008, Phi5.12, and 712 (corresponding to locus tags LPV008_gp46, Phi5.12_45 and LPV712_gp41, respectively)^28^. With the exception of phage 645, all phages were found to encode a predicted DNA polymerase subunit, while forty one of the ninety phages encode a type II orphan DNA methylase homologous to those characterised in Phi15, Phi93 and Phi145 (and which methylates the sequence 5′-CC^6m^AG-3′)^7^. Several putative homing endonuclease-encoding genes are unevenly distributed throughout the replication region though their function remains obscure. Two phages, CB13 (ORF30) and *Caseus*JM1 (ORF28) encode a putative RecJ exonuclease. HHPRED analysis indicates that this phage-encoded RecJ is similar to various 5′-3′ single stranded DNA exonucleases (GenBank: NP_417368.1, E-value = 2.9e–101), which are believed to play a role in DNA mismatch repair and homologous recombination^29^. PFAM analysis revealed the presence of a DHH domain which is common among the phosphatase family of exonucleases (PF02272.15, E-value = 4e–66). No functions could be assigned to the remaining ORFs within the replication module using either HHPRED or PFAM analysis. Interestingly, several phages contain very similar ORFs based on homology searches, although some of these homologs appear to be truncated, perhaps because their associated functions are not essential (anymore).

### Hierarchical clustering and gene complements

**Figure 6:**
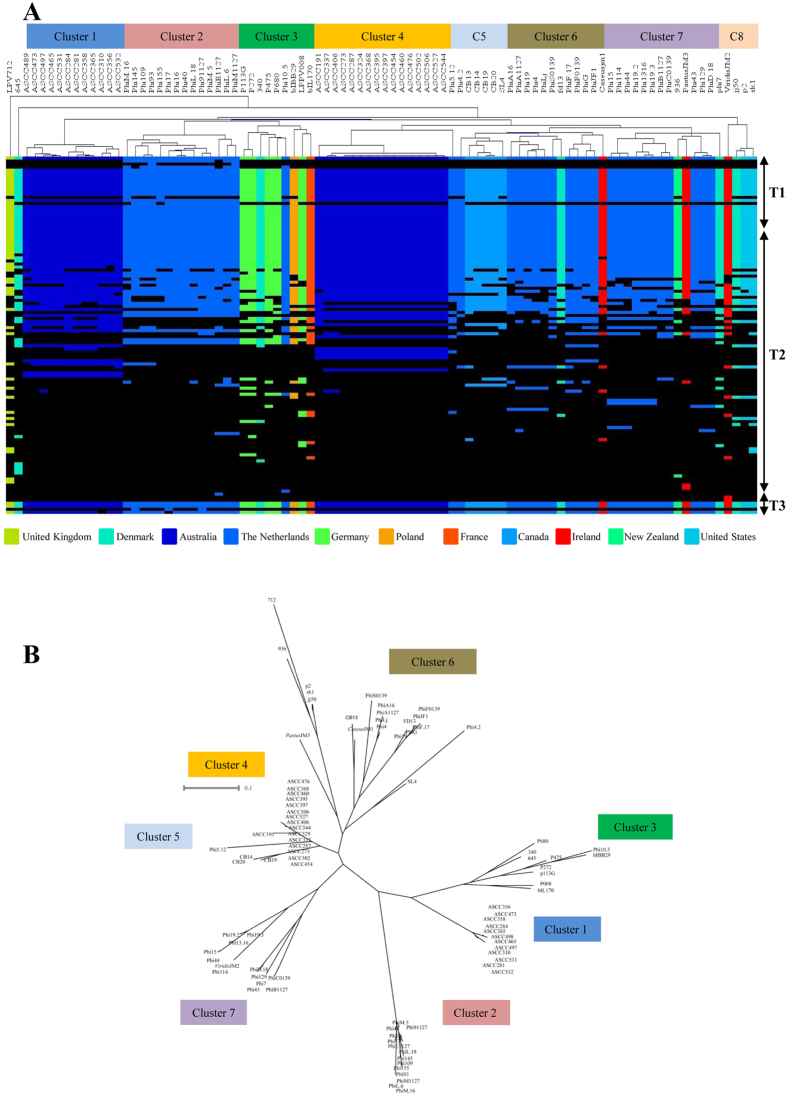
(**A**) Hierarchical clustering heatmap representing the variability of the 936 group phages. The 936 group phages appear to form eight clusters of which phage 712 and 645 are independent. Clustering was performed based on the presence (coloured squares) or absence (black squares) of a given protein family. T1, T2 and T3 denotes the three modules that correspond to the three transcripts; the late, early and middle respectively. The presence/absence heatmap was generated in Mev (V4_9_0). (**B**) ClustalW multiple genome alignment of the ninety phage genomes. Clades corresponding to the protein clusters in the HCL are highlighted.

To further scrutinise the gene product complements among the ninety 936 group phages, the BlastP all-against-all outputs (see above) were converted into a protein family presence or absence matrix, visualised using Mev4 and analysed using hierarchical clustering (HCL). The visual display of this HCL output (Fig. 6A) shows a general trend that phages isolated from the same geographical location (Country) cluster together based on their protein complement. Clusters 2, 6 and 7 harbour the phages sequenced in this study isolated from The Netherlands (Fig. 6A), while clusters 1 and 4 encompass phages isolated from Australian Dairies^3^. Furthermore, clustering of the Australian phages using HCL revealed the presence of the same subgroups as generated when employing a maximum clade credibility phylogeny, a method that has been applied previously^3^, thus signifying the usefulness of these approaches to study phage groups or populations (Fig. 6A). Exceptions to this geographical clustering are represented by phages p113G, P008, P680, P272 (Germany), which cluster together with 340 (Denmark), Phi10.5 (The Netherlands) and bIL170 (France) (Fig. 6A). The global movement of industrial starter cultures may distort the geographical clustering, particularly if the host bacterium impacts on the protein complements encoded by particular phages (e.g. by selecting for genetic adaptations to counter host-encoded phage defences). The HCL also demonstrates that the distribution of non-core protein families among the 936 group phages varies among the different clusters (Fig. 6A), substantiating the notion that the propagating host strain plays an important role in determining the phage gene complement. As discussed above, acquisition, mutation and loss of specific phage genes appears to negate certain Abi phage resistance systems. The imposed selective pressure appears to force phage populations to gain or lose certain ORFs in order to escape from these host-mediated defence systems. To determine if clustering of the 936 group phage occurs at the nucleotide level, a clustalW multiple alignment was performed on the ninety phages. As observed for the protein family-based presence or absence clustering, the 936 group phages form the same groups when clustering is based on nucleotide comparisons (Fig. 6B) with just a few exceptions, such as phages 340 and 645, which were not clustered based on protein family presence or absence (Fig. 6A), yet were linked when compared at the nucleotide level (Fig. 6B). An assessment of these two phages indicates that sequence homology is lost at the 3′ end of the early transcript in the region encoding the DNA polymerase of phage 340, and the start of the middle transcript in phage 645 (Fig. S2 A). Interestingly, phage 645 is the only 936 group phage lacking the putative DNA polymerase-encoding gene and several surrounding genes, while it apparently has gained several unique genes at the start of the middle expressed transcript. It therefore appears that phages 645 and 340 are highly related except for this region, and that this discrepancy accounts for the differential grouping of the two phages when compared at the nucleotide or protein level. To assess the nucleotide synteny of the 936 group phages, a dot plot analysis was performed on a selection of representatives from different clusters (Fig. S2 B–F). It is evident from these dot plots, that discontinuation of synteny occurs in regions we identified as sites of gene acquisition as well as regions previously predicted to be recombination hotspots. These include the packaging region, the *mcp* and *mtp* regions, the region surrounding the RBP-encoding gene, the TMP and various points within the replication region (Fig. S2 B–F), consistent with a previous report on the recombination rate and genetic diversity across representative genomes of the 936 group phages^3^. Further analysis using the multiple sequence genome alignment of the ninety phages revealed no conserved motifs that could be potential targets for recombinases (data not shown).

## Discussion

Despite several decades of scientific efforts, the 936 group phages remain the most endemic and problematic phage group in lactococcal fermentation facilities^4^. In the current study, genome sequences of a collection of 936 group phages isolated from Dutch fermentation facilities were determined so as to allow, together with previously sequenced phages, an investigation into the gene architecture of this phage group. A total of twenty nine protein families were found to be common among all sequenced 936 group phages, thus representing its core genome, which is assumed to correspond to essential functions for the production of progeny phage (DNA replication and packaging, host cell recognition and lysis as well as the formation of the phage particle^9^,^25^). The traditional theory of dsDNA phage evolution is the horizontal exchange of mosaic segments^30^, which is believed to consist of the movement of ‘functional blocks’ of core genes such as those involved in packaging, capsid and tail morphogenesis^31^, therefore accounting for the conserved syntenic nature of the structural genome in the case of the 936 phage group. Comparative phage genomics suggests that the interspersion of the non-core genes within these regions improves phage infection/propagation chances under certain conditions^13^,^32^. Our work on the 936 group phages supports the notion that the exchange of conserved functional modules leads to mosaic genomes in dsDNA phages. In the case of 936 group phages, it also demonstrates that packaging and morphogenesis modules may still exhibit substantial genetic divergence due to the presence of apparently accessory ORFs.

The function of the NPS in the 936 group phages is unknown but it is evident from this study that particular phage isolates harbour unique NPS-encoding genes which may provide certain isolates with an advantageous phenotype. One of the most striking features of the phages isolated in this study was the presence of a spiral-like structure surrounding the length of the tail of PhiC0139. Despite homologues of the *tpeX* being present in twenty one of the ninety phages, this feature appears unique to PhiC0139 and PhiB1127 (Fig. S1). Translational frameshifting plays an important role in the structural genome of several phages^18^,^33^. A +1 translational frameshift similar to the extension of the *mtp* observed for *Bacillus* phage SPP1, where this results in ‘hairy’ features on the phage tail^34^, has not previously been reported for a lactococcal phage. The presence of a carbohydrate-binding domain suggests that this additional structure is involved in host cell attachment, consistent with observations made for other dsDNA tailed phages including the ‘hairy tail’ feature on SPP1^34^,^35^. We hypothesize that this non-essential accessory ORF has been acquired to provide a competitive advantage under certain, as yet unknown, environmental conditions.

The interaction of phage RBPs to the host cell receptor is a fundamental first step in phage infection and proliferation. By correlating the phage RBP groups and the lactococcal CWPS gene cluster types we expand on the link between the receptor and anti-receptor by the identification of two novel RBP groups, designated here as IV and V. Our results indicate that the RBP-encoding gene is highly diverse to allow phages to recognize a variety of different CWPS types, and this apparent adaptability is a further indication as to why the 936 group phages represent the most dominant members of the lactococcal phages groups.

One of the most difficult aspects of characterising exclusively lytic bacteriophages is the lack of methods to study the function of hypothetical proteins through target mutational analysis and to that end the function of many proteins within the 936 genomes remain enigmatic. In an effort to understand the complexity of gene complement of the 936 group phages, a combination of MCL and HCL analysis was undertaken to identify the one hundred and eighteen protein families. As expected the majority of the core genes were associated with the late transcript. The nine fully conserved protein families that are encoded within the replication region, of which five form a single ‘module’ (Fig. 1), indicates that mosaicism is not restricted to the late transcript in the 936 group phages. Using the HCL analysis and nucleotide alignments, we demonstrated that an apparent link exists between gene complement/phylogeny and geographical origin whereby phages isolated from the same country formed clusters. Furthermore, the HCL analysis indicated that the distribution of the non-core genome is also linked to these clusters. These findings suggest that the host bacterium plays a role in the emergence of particular phages either through simple selection (e.g. RBP diversity is correlated to CWPS gene cluster types) and/or that the shuffling of genes is occurring among phage isolates in an effort to evade host resistance mechanisms. MCL- and HCL-based scrutiny demonstrates that this latter selective force is operational for those genomes in order to circumvent various host-encoded anti-phage defences, such as Abi and restriction-modification systems. Further investigations into the mode of action of as yet uncharacterised Abi systems will shed light on whether and to what extent Abi-mediated selective pressure is shaping gene composition of the replication module of 936 group phages. Finally, our study corroborates previous investigations into the 936 group phage in demonstrating that there are particular regions of the genome more prone to genetic change across all three transcripts that can be identified using nucleotide alignments but more informatively using HCL of a total gene pool.

## Materials and Methods

### Bacteria and phages

Bacterial cultures plasmids and additional phages (those not sequenced in this study but used experimentally) employed in this study are listed in Table S1; phages sequenced in this study are listed in Table 1. Seventeen phages had previously been isolated^7^,^36^. An additional twenty one phages from whey samples collected in 2013 from two of the three previously employed fermentation facilities (F1 and F3), and from four whey cream samples from a fourth Dutch dairy factory (F4, unknown starter culture). Phages were propagated on their corresponding host(s) as described previously^36^.

### GenBank accession numbers

The GenBank accession numbers for the phages sequenced in this study are listed in Table 1. The accession numbers for all previously sequenced lactococcal 936 group phages can be obtained from the European Bioinformatics Institute (http://www.ebi.ac.uk/genomes/phage.html).

### Phage genome sequencing and annotations

DNA was isolated from the thirty five phages listed in Table 1 and whole genome sequencing performed on a Roche 454 FLX titanium instrument (Macrogen Inc., Korea) (average of 220-fold sequence coverage) was carried out as described previously^7^. Sequencing across the cohesive ends of the genomes was achieved by ligating phage DNA prior to Sanger sequencing of the 5′ and 3′ ligated ends. Open reading frames (ORFs) were automatically predicted using GeneMark^37^. General feature format (gff) files were generated of the predicted phage proteomes and visualised using the annotation software, Artemis, v10^38^. ORF boundaries were verified or, if necessary, adjusted by manual inspection of Shine-Dalgarno sequences. BlastP^39^ was employed to assist in functional annotation of the predicted gene products.

### Defining the core and variable 936 genome

To determine the core and variable genome of the 936 group phages, all publically available 936 group phage genome sequences were retrieved from the GenBank database. To ensure accurate determination of the core genome, the gene annotation criteria used for the phage genomes from this study were also applied to previously sequenced phages to ensure that ORF identification methods had been applied consistently. Sequence comparisons at the protein level of a total of ninety 936 group phage genomes was carried out using an all-against-all bi-directional BLAST alignment^39^ (cut-off: *E*-value 0.0001, with at least 50% identity across at least 50% of either protein sequence), and the resulting output was then clustered into protein families sharing the same function using MCL implemented in the mclblastline pipeline v12-0678^40^. Phylogenetic trees were generated using Mega (V6.0)^41^.

To perform the Hierarchical clustering, the output from the BLASTP-based MCL clustering was used to perform a two-way hierarchical clustering, in order to group phage genomes based on their similarity at protein level. A binary matrix containing presence/absence of each MCL family in all ninety phages was first computed and used as input of MeV suite (V4.9)^42^, which performed the hierarchical clustering. An additional clustalW^43^ multiple genome alignment of the ninety phage genomes was also performed based on the full genome length at nucleotide level. Dot plot analysis was performed using Gepard^44^.

### Phage gene cloning and protein expression

For the construction of the derivative of pTX8048, designated pTX15_NPS, and the derivative of pNZ8048, designated pNZtpeX, DNA fragments encompassing the entire NPS-encoding gene from Phi15, designated *npsPhi15* (corresponding to locus tag Phi15_12), and a DNA fragment encoding a putative tail protein extension from PhiC0139, designated *tpeX*, (corresponding to locus tag PhiC0139_13), was generated by PCR using the primers listed in Table S1 and employing KOD high fidelity polymerase (Millipore, Cork, Ireland). Following overnight double digestion of the plasmids and amplicons with relevant restriction endonucleases (Table S1), overnight ligation (T4 DNA ligase, NEB, MA, USA) was performed to generate pTX15_NPS and pNZtpeX. The resulting ligation mixture was introduced into *L. lactis* NZ9000 by electroporation and transformants were then selected on M17 agar plates supplemented with 0.5% glucose (GM17) and 10 μg ml^−1^ chloramphenicol (Sigma) at 30 °C. Sanger sequencing was employed to verify the sequence integrity of the generated constructs (MWG Eurofins, Germany) using primers mcsPTX8048F and mcsPTX8048R for pTX15_NPS, and C0139tpex_F and mcsPTX8048R for pNZtpeX (Table S1). Heterologous gene expression using *L. lactis* NZ9000 harbouring pTX15_NPS or pNZtpeX was performed using the Nisin Inducible Expression (NICE) and Thioredoxin (Trx) fusion system incorporating a hexahistidine (His) tag for purification as described previously^45^,^46^. Protein purification of His-tagged Phi15-NPS or TpeX using a Ni-NTA agarose column (Qiagen, Manchester, United Kingdom) was performed as described by previously^46^. The samples were run on 12% SDS-PAGE gels to confirm the presence of induced target proteins^46^. The protein concentration of each fraction was quantified using the standard BioRad protein (BSA) assay (BioRad, Dublin, Ireland)^47^.

### Polyclonal antibody preparation, Electron microscopy, SDS-PAGE and Western Hybridisation

Immunogold electron microscopy was performed as described previously with polyclonal antibodies directed against Phi15-NPS, TpeX or phage p2 MTP^48^ developed by Davids Biotechnologie (Regensburg, Germany) using their standard protocol. Purified bacteriophage suspensions obtained from CsCl were dialysed against SM buffer (50 mM Tris-HCL, 100 mM NaCl and 8 mM MgSO_4_, pH 6.5, Sigma). Staining was performed with 2% (w/v) uranyl acetate on freshly prepared carbon films. Grids were analysed in a Tecnai 10 transmission electron microscope (TEM) (FEI Company, Eindhoven, The Netherlands, OR, USA) at an acceleration voltage of 80 kV. Micrographs were taken with a MegaView II charge-coupled device camera at the Max Rubner-Institut, Kiel, Germany^48^,^49^,^50^,^51^,^52^. Phage proteins, from CsCl-purified phage particles, were separated on a 12% SDS-PAGE gel. Western hybridisation was performed as outlined previously^45^,^46^ using polyclonal antibodies raised against Phi15-NPS, TpeX or phage p2 MTP.

## Additional Information

**How to cite this article**: Murphy, J. *et al.* Comparative genomics and functional analysis of the 936 group of lactococcal *Siphoviridae* phages. *Sci. Rep.* **6**, 21345; doi: 10.1038/srep21345 (2016).

## Supplementary Material

Supplementary Information

## Figures and Tables

**Table 1:** The collection of 936 group phages reported in this study.

Factory	Year isolated	Phage	No of ORFs	Genome size (bp)	*Cos*site	Structural classification	RBP Group	GenBank accession number
F1	2009	Phi43	54	29 420	CACAAAGGACT	V3	I	KP793110
F1	2009	Phi19	50	28 437	CACAAAGGACT	V3	IV	KP793103
F1	2009	Phi4	51	28 767	CACAAAGGACT	V3	IV	KP793101
F1	2009	Phi17	56	30 463	CACAAAGGTCT	V2	IV	KP793114
F1	2009	PhiJF1	58	31 901	CACAAAGGACT	V3	I	KP793129
F1	2009	PhiG	58	30 596	CACAAAGGACT	V3	I	KP793117
F1	2013	PhiA.16	49	28 349	CACAAAGGACT	V3	IV	KP793102
F1	2013	PhiD.18	53	28 833	CACAGAGGACT	V3	I	KP793107
F1	2013	Phi13.16	54	30 577	CACAAAGGACT	V3	I	KP793116
F1	2013	PhiL.18	55	30 882	CACAAAGGTCT	V2	IV	KP793120
F1	2013	PhiF.17	56	30 389	CACAAAGGACT	V3	I	KP793113
F1	2013	Phi5.12	53	29 026	CACAAAGGACT	V2	I	KP793108
F1	2013	PhiM.16	54	31 869	CACAAAGGTCT	V3	IV	KP793128
F2	2009	Phi109	54	31 033	CACAAAGGTCT	V2	IV	KP793121
F2	2009	Phi93	55	31 825	CACAAAGGTCT	V2	IV	KM091443
F2	2009	Phi129	54	30 178	CACAAAGGTCT	V3	I	KP793112
F2	2009	PhiLj	49	29 983	CACAAAGGACT	V3	IV	KP793133
F3	2009	Phi155	55	32 150	CACAAAGGTCT	V3	IV	KP793130
F3	2009	Phi16	57	31 561	CACAAAGGACT	V2	IV	KP793135
F3	2009	Phi44	52	31 379	CACAAAGGACT	V3	I	KP793124
F3	2009	Phi114	53	30 532	CACAAAGGACT	V3	I	KP793115
F3	2009	Phi15	55	31 946	CACAAAGGACT	V3	I	KM091442
F3	2009	Phi40	57	31 631	CACAAAGGTCT	V2	IV	KP793127
F3	2009	Phi145	55	30 861	CACAAAGGTCT	V2	IV	KM091444
F3	2013	Phi10.5	63	31 805	CACAAAGGGCT	V1	III	KP793119
F3	2013	Phi19.3	52	28 811	CACAAAGGACT	V3	I	KP793105
F3	2013	Phi19.2	55	29 984	CACAAAGGACT	V3	I	KP793111
F3	2013	PhiL.6	59	31 181	CACAAAGGACT	V2	IV	KP793122
F3	2013	Phi4.2	59	31 375	CACAAAGGACT	V5	I (ORF21)	KP793123
F3	2013	PhiM.5	58	31 498	CACAAAGGTCT	V2	IV	KP793126
F4	2013	PhiF0139	57	30 800	CACAAAGGACT	V3	I	KP793118
F4	2013	PhiA1127	52	28 873	CACAAAGGACT	V3	IV	KP793106
F4	2013	PhiC0139	53	29 032	CACAAAGGACT	V4	V	KP793109
F4	2013	Phi91127	58	31 495	CACAAAGGTCT	V2	IV	KP793125
F4	2013	PhiB1127	53	28 785	CACAAAGGACT	V4	V	KP793104
F4	2013	PhiS0139	53	30 120	CACAAAGGACT	V3	I	KP793134
F4	2013	PhiE1127	60	32 270	CACAAAGGTCT	V2	IV	KP793131
F4	2013	PhiM1127	59	32 682	CACAAAGGTCT	V2	IV	KP793132
