# Supplementary material for: Comparative genomics and functional analysis of the 936 group of lactococcal Siphoviridae phages
Source: Sci Rep. 2016 Feb 19;6:21345. doi: 10.1038/srep21345 (PMC4759559; doi:10.1038/srep21345)
Supplement: Supplementary Information [file srep21345-s1.pdf]

Title: Comparative genomics and functional analysis of the 936 group of lactococcal *Siphoviridae* phages

Authors: James Murphy[1], Francesca Bottacini[2], Jennifer Mahony[1], Philip Kelleher[1], Horst Neve[3], Aldert Zomer[1,2,*], Arjen Nauta[4] and Douwe van Sinderen[1,2,#].

[1]School of Microbiology, University College Cork, Cork, Ireland,
[2]Alimentary Pharmabiotic Centre, University College Cork, Cork, Ireland,
[3]Department of Microbiology and Biotechnology, Max Rubner Institut, Kiel, Germany
[4]FrieslandCampina, Amersfoort, The Netherlands.

Figure S1. Amino acid sequence alignment of the TpeX homologs in the 936 group phages. Differences in amino acid sequences in comparison to PhiC0139 are shaded black. Potential amino acids involved in carbohydrate binding as determined by HHPRED are highlighted in the red boxes.

Figure S2. Dotplot analysis of a selection of 936 group phages. Sites were loss of synteny correlate with sites of gene arrangements are highlighted by black arrows. **A)** 340 vs 645. **B)** P008 vs PhiA.16. **C)** ASCC532 vs P008. **D)** PhiM.16 vs ASCC532. **E)** Phi19.2 vs PhiM.16 and **F)** Phi19.2 vs Phi5.12

Figure S1

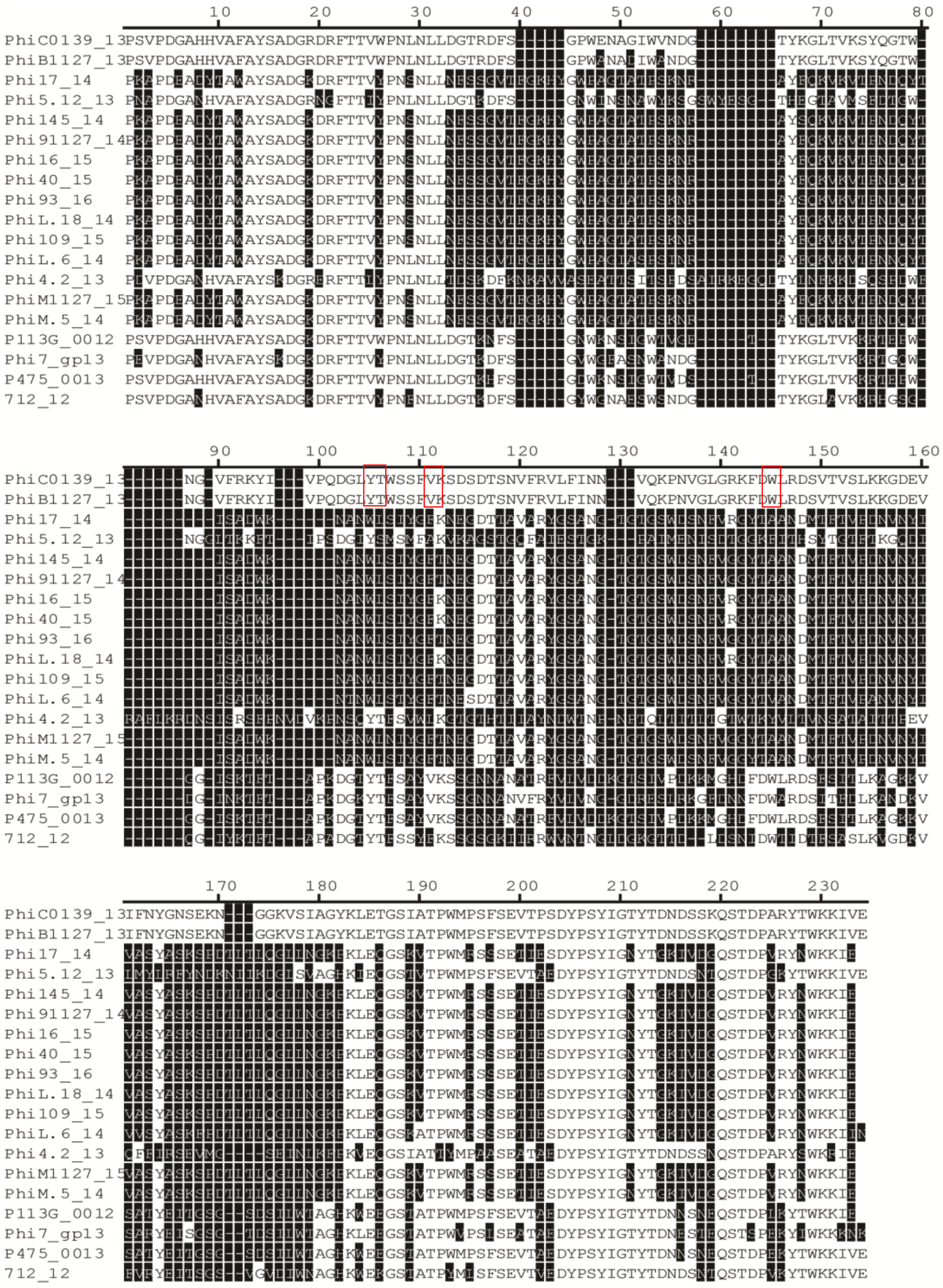

Figure S2

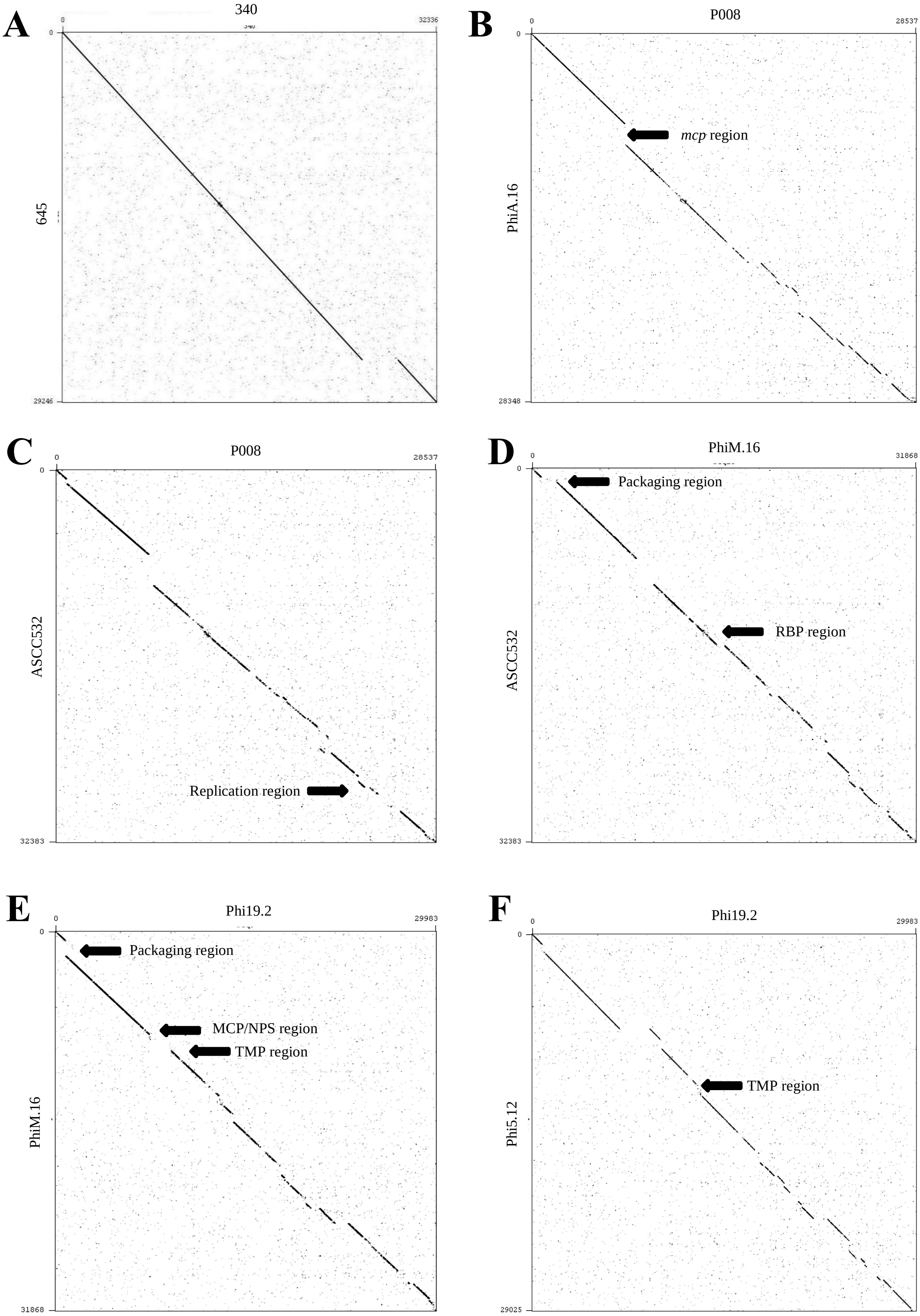
A
340
0
32336
0
645
29246
B
P008
0
28537
0
PhiA.16
28348
mcp region
C
P008
0
28537
0
ASCC532
32383
Replication region
D
PhiM.16
0
31868
0
ASCC532
32383
Packaging region
RBP region
E
Phi19.2
0
29983
0
PhiM.16
31868
Packaging region
MCP/NPS region
TMP region
F
Phi19.2
0
29983
0
Phi5.12
29025
TMP region

Table S1. Bacteria, plasmids, additional phages and primers used in this study.

Lactococcal subspecies	Strain (CWPS type)	Source
ssp. *lactis*	SM A (Unknown)	36
ssp. *cremoris*	SM B (A)	36
ssp. *cremoris*	SM C (A)	36
ssp. *cremoris*	SM D (Unknown)	36
ssp. *lactis* biovar *diacetylactis*	SM E (Unknown)	36
ssp. *lactis*	SM F (Unknown)	36
ssp. *cremoris*	SM G (Unknown)	36
ssp. *cremoris*	SM H (C)	36
ssp. *cremoris*	SM I (C)	36
ssp. *cremoris*	SM J (C)	36
ssp. *cremoris*	SM K (Unknown)	36
ssp. *lactis* biovar *diacetylactis*	SM L (Unknown)	36
ssp. *lactis* biovar *diacetylactis*	SM M (Unknown)	36
ssp. *lactis*	SM N (C)	36
ssp. *cremoris*	SM O (C)	36
ssp. *lactis*	SM P (C)	36
ssp. *cremoris*	SM Q (C)	36
ssp. *cremoris*	SM R (C)	36
ssp. *cremoris*	SM S (C)	36
ssp. *cremoris*	SM T (C)	36
ssp. *cremoris*	SM 1 (C)	36
ssp. *lactis* biovar *diacetylactis*	SM 2 (B)	36
ssp. *cremoris*	SM 3 (Unknown)	36
ssp. *cremoris*	SM 4 (C)	36
ssp. *lactis* biovar *diacetylactis*	SM 5 (C)	36
ssp. *lactis* biovar *diacetylactis*	SM 6 (B)	36
ssp. *cremoris*	SM 7 (C)	36
ssp. *lactis* biovar *diacetylactis*	SM 8 (Unknown)	36
ssp. *lactis* biovar *diacetylactis*	SM 9 (Unknown)	36
ssp. *cremoris*	SM 10 (B)	36
ssp. *cremoris*	SM 11 (C)	36
ssp. *lactis*	SM 12 (Unknown)	36
ssp. *lactis*	SM 13 (C)	36
ssp. *lactis* biovar *diacetylactis*	SM 14 (B)	36
ssp. *lactis*	SM 15 (C)	36
ssp. *lactis* biovar *diacetylactis*	SM 16 (Unknown)	36
ssp. *lactis* biovar *diacetylactis*	SM 17 (B)	36
ssp. *cremoris*	SM 18 (C)	36
ssp. *cremoris*	SM 19 (C)	36
ssp. *cremoris*	SM 20 (Unknown)	36
ssp. *cremoris*	UC509.9 (A). Host for Tuc2009	49
ssp. *cremoris*	158 (A). Host for P1084	50
ssp. *lactis*	IL1403 (B). Host for P008	51
**Additional phages**	**Features**	
Tuc2009	P335 group phage. Negative control for Phi15-NPS	49

P1084	936 group phage with spiral like decorations. Genome not sequenced	Personal communication
P008	936 group phage. Negative control for TpxE antibodies	[52]
**Plasmids**	**Features**	**Source**
pTX8048	Chloramphenicol resistant	[45]
pTX15_NPS	pTX8048 derivative encoding *npsPhi15*	This study
pNZtpeX	pNZ8048 derivative encoding *fiberPhiC0139*	This study
**Primers**	**Features**	**Source**
mcsPTX8048 F	TAACCTGGCCGGTTCTGGTTC	This study
mcsPTX8048 R	CGTTTCAAGCCTTGGTTTTC	This study
Phi15_NPSF	aaattt**GGATCC**ATGAGTTTAGATAATTTTAG	This study
Phi15_NPSR	aaattt**TCTAGA**CTAATCAATGAAATAGCTTG	This study
C0139tpeX_F	agcagc**CCATGG**cc*CACCATCACCATCACCAT*tcttctggt GACGGGGCTCACCACG[c]	This study
C0139 tpeX _R	agcagc**TCTAGA**TTATTCTACTATTTTTTTCCAGGTATATC	This study

Text file 1. The core genes of the 936 group phage and their predicted function

Protein Function	ID
Small Terminase	340_001
Small Terminase	645_001
Small Terminase	936_001
Small Terminase	ASCC191_0001
Small Terminase	ASCC273_0001
Small Terminase	ASCC281_0001
Small Terminase	ASCC284_0001
Small Terminase	ASCC287_0001
Small Terminase	ASCC310_0001
Small Terminase	ASCC324_0001
Small Terminase	ASCC337_0001
Small Terminase	ASCC356_0001
Small Terminase	ASCC358_0001
Small Terminase	ASCC365_0001
Small Terminase	ASCC368_0001
Small Terminase	ASCC395_0001
Small Terminase	ASCC397_0001
Small Terminase	ASCC406_0001
Small Terminase	ASCC454_0001
Small Terminase	ASCC460_0001
Small Terminase	ASCC465_0001
Small Terminase	ASCC473_0001
Small Terminase	ASCC476_0001
Small Terminase	ASCC489_0001
Small Terminase	ASCC497_0001
Small Terminase	ASCC502_0001
Small Terminase	ASCC506_0001
Small Terminase	ASCC527_0001
Small Terminase	ASCC531_0001
Small Terminase	ASCC532_0001
Small Terminase	ASCC544_0001
Small Terminase	CB13_0001
Small Terminase	CB14_0001
Small Terminase	CB19_0001
Small Terminase	CB20_0001
Small Terminase	LPPV008_gp01
Small Terminase	LPV712_gp001
Small Terminase	P113G_001
Small Terminase	P272_001
Small Terminase	P475_001
Small Terminase	P680_01
Small Terminase	PastusJM3_01
Small Terminase	Phi10.5_01
Small Terminase	Phi109_01
Small Terminase	Phi114_01
Small Terminase	Phi129_01
Small Terminase	Phi1316_01
Small Terminase	Phi145_01

182	Small Terminase	Phi155_01
183	Small Terminase	Phi15_01
184	Small Terminase	Phi16_01
185	Small Terminase	Phi17_01
186	Small Terminase	Phi19.2_01
187	Small Terminase	Phi19.3_01
188	Small Terminase	Phi19_01
189	Small Terminase	Phi4.2_01
190	Small Terminase	Phi40_01
191	Small Terminase	Phi43_01
192	Small Terminase	Phi44_01
193	Small Terminase	Phi4_01
194	Small Terminase	Phi5.12_01
195	Small Terminase	Phi91127_01
196	Small Terminase	Phi93_01
197	Small Terminase	PhiA1127_01
198	Small Terminase	PhiA16_01
199	Small Terminase	PhiB1127_01
200	Small Terminase	PhiC0139_01
201	Small Terminase	PhiD.18_01
202	Small Terminase	PhiE1127_01
203	Small Terminase	PhiF.17_01
204	Small Terminase	PhiF0139_01
205	Small Terminase	PhiG_01
206	Small Terminase	PhiJF1_01
207	Small Terminase	PhiL.18_01
208	Small Terminase	PhiL.6_01
209	Small Terminase	PhiLj_01
210	Small Terminase	PhiM.16_01
211	Small Terminase	PhiM.5_01
212	Small Terminase	PhiM1127_01
213	Small Terminase	PhiS0139_01
214	Small Terminase	SL4_0001
215	Small Terminase	ViridisJM2_gp01
216	Small Terminase	bIBB29_gp01
217	Small Terminase	bIL170p01
218	Small Terminase	fd13_001
219	Small Terminase	jj50_ORF1
220	Small Terminase	jm1_0001
221	Small Terminase	p2_0001
222	Small Terminase	phi7_01
223	Small Terminase	sk1p01
224	Large terminase	340_002
225	Large terminase	645_002
226	Large terminase	936_002
227	Large terminase	ASCC191_0003
228	Large terminase	ASCC273_0003
229	Large terminase	ASCC281_0004
230	Large terminase	ASCC284_0004
231	Large terminase	ASCC287_0003

232	Large terminase	ASCC310_0004
233	Large terminase	ASCC324_0003
234	Large terminase	ASCC337_0003
235	Large terminase	ASCC356_0004
236	Large terminase	ASCC358_0004
237	Large terminase	ASCC365_0004
238	Large terminase	ASCC368_0003
239	Large terminase	ASCC395_0003
240	Large terminase	ASCC397_0003
241	Large terminase	ASCC406_0003
242	Large terminase	ASCC454_0003
243	Large terminase	ASCC460_0003
244	Large terminase	ASCC465_0004
245	Large terminase	ASCC473_0004
246	Large terminase	ASCC476_0003
247	Large terminase	ASCC489_0004
248	Large terminase	ASCC497_0004
249	Large terminase	ASCC502_0003
250	Large terminase	ASCC506_0003
251	Large terminase	ASCC527_0003
252	Large terminase	ASCC531_0004
253	Large terminase	ASCC532_0004
254	Large terminase	ASCC544_0003
255	Large terminase	CB13_0003
256	Large terminase	CB14_0003
257	Large terminase	CB19_0003
258	Large terminase	CB20_0003
259	Large terminase	LPPV008_gp02
260	Large terminase	LPV712_gp002
261	Large terminase	P113G_002
262	Large terminase	P272_002
263	Large terminase	P475_002
264	Large terminase	P680_02
265	Large terminase	PastusJM3_02
266	Large terminase	Phi10.5_03
267	Large terminase	Phi109_04
268	Large terminase	Phi114_03
269	Large terminase	Phi129_02
270	Large terminase	Phi1316_02
271	Large terminase	Phi145_03
272	Large terminase	Phi155_04
273	Large terminase	Phi15_03
274	Large terminase	Phi16_04
275	Large terminase	Phi17_04
276	Large terminase	Phi19.2_02
277	Large terminase	Phi19.3_02
278	Large terminase	Phi19_02
279	Large terminase	Phi4.2_03
280	Large terminase	Phi40_04
281	Large terminase	Phi43_02

282	Large terminase	Phi44_03
283	Large terminase	Phi4_02
284	Large terminase	Phi5.12_03
285	Large terminase	Phi91127_04
286	Large terminase	Phi93_05
287	Large terminase	PhiA1127_03
288	Large terminase	PhiA16_02
289	Large terminase	PhiB1127_02
290	Large terminase	PhiC0139_02
291	Large terminase	PhiD.18_02
292	Large terminase	PhiE1127_03
293	Large terminase	PhiF.17_03
294	Large terminase	PhiF0139_03
295	Large terminase	PhiG_03
296	Large terminase	PhiJF1_03
297	Large terminase	PhiL.18_04
298	Large terminase	PhiL.6_04
299	Large terminase	PhiLj_02
300	Large terminase	PhiM.16_04
301	Large terminase	PhiM.5_04
302	Large terminase	PhiM1127_05
303	Large terminase	PhiS0139_02
304	Large terminase	SL4_0003
305	Large terminase	ViridisJM2_gp02
306	Large terminase	bIBB29_gp02
307	Large terminase	bIL170p02
308	Large terminase	fd13_002
309	Large terminase	jj50_ORF2
310	Large terminase	jm1_0002
311	Large terminase	p2_0002
312	Large terminase	phi7_02
313	Large terminase	sk1p02
314	HNH Endonuclease	340_003
315	HNH Endonuclease	645_003
316	HNH Endonuclease	936_003
317	HNH Endonuclease	ASCC191_0004
318	HNH Endonuclease	ASCC273_0004
319	HNH Endonuclease	ASCC281_0005
320	HNH Endonuclease	ASCC284_0005
321	HNH Endonuclease	ASCC287_0004
322	HNH Endonuclease	ASCC310_0005
323	HNH Endonuclease	ASCC324_0004
324	HNH Endonuclease	ASCC337_0004
325	HNH Endonuclease	ASCC356_0005
326	HNH Endonuclease	ASCC358_0005
327	HNH Endonuclease	ASCC365_0005
328	HNH Endonuclease	ASCC368_0004
329	HNH Endonuclease	ASCC395_0004
330	HNH Endonuclease	ASCC397_0004
331	HNH Endonuclease	ASCC406_0004

332	HNH Endonuclease	ASCC454_0004
333	HNH Endonuclease	ASCC460_0004
334	HNH Endonuclease	ASCC465_0005
335	HNH Endonuclease	ASCC473_0005
336	HNH Endonuclease	ASCC476_0004
337	HNH Endonuclease	ASCC489_0005
338	HNH Endonuclease	ASCC497_0005
339	HNH Endonuclease	ASCC502_0004
340	HNH Endonuclease	ASCC506_0004
341	HNH Endonuclease	ASCC527_0004
342	HNH Endonuclease	ASCC531_0005
343	HNH Endonuclease	ASCC532_0005
344	HNH Endonuclease	ASCC544_0004
345	HNH Endonuclease	CB13_0004
346	HNH Endonuclease	CB14_0004
347	HNH Endonuclease	CB19_0004
348	HNH Endonuclease	CB20_0004
349	HNH Endonuclease	LPPV008_gp03
350	HNH Endonuclease	LPV712_gp003
351	HNH Endonuclease	P113G_003
352	HNH Endonuclease	P272_003
353	HNH Endonuclease	P475_003
354	HNH Endonuclease	P680_03
355	HNH Endonuclease	PastusJM3_03
356	HNH Endonuclease	Phi10.5_04
357	HNH Endonuclease	Phi109_05
358	HNH Endonuclease	Phi114_04
359	HNH Endonuclease	Phi129_03
360	HNH Endonuclease	Phi1316_03
361	HNH Endonuclease	Phi145_04
362	HNH Endonuclease	Phi155_05
363	HNH Endonuclease	Phi15_04
364	HNH Endonuclease	Phi16_05
365	HNH Endonuclease	Phi17_05
366	HNH Endonuclease	Phi19.2_03
367	HNH Endonuclease	Phi19.3_03
368	HNH Endonuclease	Phi19_03
369	HNH Endonuclease	Phi4.2_04
370	HNH Endonuclease	Phi40_05
371	HNH Endonuclease	Phi43_03
372	HNH Endonuclease	Phi44_04
373	HNH Endonuclease	Phi4_03
374	HNH Endonuclease	Phi5.12_04
375	HNH Endonuclease	Phi91127_05
376	HNH Endonuclease	Phi93_06
377	HNH Endonuclease	PhiA1127_04
378	HNH Endonuclease	PhiA16_03
379	HNH Endonuclease	PhiB1127_03
380	HNH Endonuclease	PhiC0139_03
381	HNH Endonuclease	PhiD.18_03

382	HNH Endonuclease	PhiE1127_04
383	HNH Endonuclease	PhiF.17_04
384	HNH Endonuclease	PhiF0139_04
385	HNH Endonuclease	PhiG_04
386	HNH Endonuclease	PhiJF1_04
387	HNH Endonuclease	PhiL.18_05
388	HNH Endonuclease	PhiL.6_05
389	HNH Endonuclease	PhiLj_03
390	HNH Endonuclease	PhiM.16_05
391	HNH Endonuclease	PhiM.5_05
392	HNH Endonuclease	PhiM1127_06
393	HNH Endonuclease	PhiS0139_03
394	HNH Endonuclease	SL4_0004
395	HNH Endonuclease	ViridisJM2_gp03
396	HNH Endonuclease	bIBB29_gp03
397	HNH Endonuclease	bIL170p03
398	HNH Endonuclease	fd13_003
399	HNH Endonuclease	jj50_ORF3
400	HNH Endonuclease	jm1_0003
401	HNH Endonuclease	p2_0003
402	HNH Endonuclease	phi7_03
403	HNH Endonuclease	sk1p03
404	Portal	340_004
405	Portal	645_004
406	Portal	936_004
407	Portal	ASCC191_0005
408	Portal	ASCC273_0005
409	Portal	ASCC281_0006
410	Portal	ASCC284_0006
411	Portal	ASCC287_0005
412	Portal	ASCC310_0006
413	Portal	ASCC324_0005
414	Portal	ASCC337_0005
415	Portal	ASCC356_0006
416	Portal	ASCC358_0006
417	Portal	ASCC365_0006
418	Portal	ASCC368_0005
419	Portal	ASCC395_0005
420	Portal	ASCC397_0005
421	Portal	ASCC406_0005
422	Portal	ASCC454_0005
423	Portal	ASCC460_0005
424	Portal	ASCC465_0006
425	Portal	ASCC473_0006
426	Portal	ASCC476_0005
427	Portal	ASCC489_0006
428	Portal	ASCC497_0006
429	Portal	ASCC502_0005
430	Portal	ASCC506_0005
431	Portal	ASCC527_0005

432	Portal	ASCC531_0006
433	Portal	ASCC532_0006
434	Portal	ASCC544_0005
435	Portal	CB13_0005
436	Portal	CB14_0005
437	Portal	CB19_0005
438	Portal	CB20_0005
439	Portal	LPPV008_gp04
440	Portal	LPV712_gp004
441	Portal	P113G_004
442	Portal	P272_004
443	Portal	P475_004
444	Portal	P680_04
445	Portal	PastusJM3_04
446	Portal	Phi10.5_05
447	Portal	Phi109_06
448	Portal	Phi114_05
449	Portal	Phi129_04
450	Portal	Phi1316_04
451	Portal	Phi145_05
452	Portal	Phi155_06
453	Portal	Phi15_05
454	Portal	Phi16_06
455	Portal	Phi17_06
456	Portal	Phi19.2_04
457	Portal	Phi19.3_04
458	Portal	Phi19_04
459	Portal	Phi4.2_05
460	Portal	Phi40_06
461	Portal	Phi43_04
462	Portal	Phi44_05
463	Portal	Phi4_04
464	Portal	Phi5.12_05
465	Portal	Phi91127_06
466	Portal	Phi93_07
467	Portal	PhiA1127_05
468	Portal	PhiA16_04
469	Portal	PhiB1127_04
470	Portal	PhiC0139_04
471	Portal	PhiD.18_04
472	Portal	PhiE1127_05
473	Portal	PhiF.17_05
474	Portal	PhiF0139_05
475	Portal	PhiG_05
476	Portal	PhiJF1_05
477	Portal	PhiL.18_06
478	Portal	PhiL.6_06
479	Portal	PhiLj_04
480	Portal	PhiM.16_06
481	Portal	PhiM.5_06

482	Portal	PhiM1127_07
483	Portal	PhiS0139_04
484	Portal	SL4_0005
485	Portal	ViridisJM2_gp04
486	Portal	bIBB29_gp04
487	Portal	bIL170p04
488	Portal	fd13_004
489	Portal	jj50_ORF4
490	Portal	jm1_0004
491	Portal	p2_0004
492	Portal	phi7_04
493	Portal	sk1p04
494	Protease	340_005
495	Protease	645_005
496	Protease	936_005
497	Protease	ASCC191_0006
498	Protease	ASCC273_0006
499	Protease	ASCC281_0007
500	Protease	ASCC284_0007
501	Protease	ASCC287_0006
502	Protease	ASCC310_0007
503	Protease	ASCC324_0006
504	Protease	ASCC337_0006
505	Protease	ASCC356_0007
506	Protease	ASCC358_0007
507	Protease	ASCC365_0007
508	Protease	ASCC368_0006
509	Protease	ASCC395_0006
510	Protease	ASCC397_0006
511	Protease	ASCC406_0006
512	Protease	ASCC454_0006
513	Protease	ASCC460_0006
514	Protease	ASCC465_0007
515	Protease	ASCC473_0007
516	Protease	ASCC476_0006
517	Protease	ASCC489_0007
518	Protease	ASCC497_0007
519	Protease	ASCC502_0006
520	Protease	ASCC506_0006
521	Protease	ASCC527_0006
522	Protease	ASCC531_0007
523	Protease	ASCC532_0007
524	Protease	ASCC544_0006
525	Protease	CB13_0006
526	Protease	CB14_0006
527	Protease	CB19_0006
528	Protease	CB20_0006
529	Protease	LPPV008_gp05
530	Protease	LPV712_gp005
531	Protease	P113G_005

532	Protease	P272_005
533	Protease	P475_005
534	Protease	P680_05
535	Protease	PastusJM3_05
536	Protease	Phi10.5_06
537	Protease	Phi109_07
538	Protease	Phi114_06
539	Protease	Phi129_05
540	Protease	Phi1316_05
541	Protease	Phi145_06
542	Protease	Phi155_07
543	Protease	Phi15_06
544	Protease	Phi16_07
545	Protease	Phi17_07
546	Protease	Phi19.2_05
547	Protease	Phi19.3_05
548	Protease	Phi19_05
549	Protease	Phi4.2_06
550	Protease	Phi40_07
551	Protease	Phi43_05
552	Protease	Phi44_06
553	Protease	Phi4_05
554	Protease	Phi5.12_06
555	Protease	Phi91127_07
556	Protease	Phi93_08
557	Protease	PhiA1127_06
558	Protease	PhiA16_05
559	Protease	PhiB1127_05
560	Protease	PhiC0139_05
561	Protease	PhiD.18_05
562	Protease	PhiE1127_06
563	Protease	PhiF.17_06
564	Protease	PhiF0139_06
565	Protease	PhiG_06
566	Protease	PhiJF1_06
567	Protease	PhiL.18_07
568	Protease	PhiL.6_07
569	Protease	PhiLj_05
570	Protease	PhiM.16_07
571	Protease	PhiM.5_07
572	Protease	PhiM1127_08
573	Protease	PhiS0139_05
574	Protease	SL4_0006
575	Protease	ViridisJM2_gp05
576	Protease	bIBB29_gp05
577	Protease	bIL170p05
578	Protease	fd13_005
579	Protease	jj50_ORF5
580	Protease	jm1_0005
581	Protease	p2_0005

582	Protease	phi7_05
583	Protease	sk1p05
584	Major capsid protein	340_007
585	Major capsid protein	645_006
586	Major capsid protein	936_006
587	Major capsid protein	ASCC191_0007
588	Major capsid protein	ASCC273_0007
589	Major capsid protein	ASCC281_0008
590	Major capsid protein	ASCC284_0008
591	Major capsid protein	ASCC287_0007
592	Major capsid protein	ASCC310_0008
593	Major capsid protein	ASCC324_0007
594	Major capsid protein	ASCC337_0007
595	Major capsid protein	ASCC356_0008
596	Major capsid protein	ASCC358_0008
597	Major capsid protein	ASCC365_0008
598	Major capsid protein	ASCC368_0007
599	Major capsid protein	ASCC395_0007
600	Major capsid protein	ASCC397_0007
601	Major capsid protein	ASCC406_0007
602	Major capsid protein	ASCC454_0007
603	Major capsid protein	ASCC460_0007
604	Major capsid protein	ASCC465_0008
605	Major capsid protein	ASCC473_0008
606	Major capsid protein	ASCC476_0007
607	Major capsid protein	ASCC489_0008
608	Major capsid protein	ASCC497_0008
609	Major capsid protein	ASCC502_0007
610	Major capsid protein	ASCC506_0007
611	Major capsid protein	ASCC527_0007
612	Major capsid protein	ASCC531_0008
613	Major capsid protein	ASCC532_0008
614	Major capsid protein	ASCC544_0007
615	Major capsid protein	CB13_0007
616	Major capsid protein	CB14_0007
617	Major capsid protein	CB19_0007
618	Major capsid protein	CB20_0007
619	Major capsid protein	LPPV008_gp06
620	Major capsid protein	LPPV008_gp07
621	Major capsid protein	LPV712_gp006
622	Major capsid protein	P113G_006
623	Major capsid protein	P272_006
624	Major capsid protein	P475_006
625	Major capsid protein	P680_06
626	Major capsid protein	PastusJM3_06
627	Major capsid protein	Phi10.5_07
628	Major capsid protein	Phi109_08
629	Major capsid protein	Phi114_07
630	Major capsid protein	Phi129_06
631	Major capsid protein	Phi1316_06

632	Major capsid protein	Phi145_07
633	Major capsid protein	Phi155_08
634	Major capsid protein	Phi15_07
635	Major capsid protein	Phi16_08
636	Major capsid protein	Phi17_08
637	Major capsid protein	Phi19.2_06
638	Major capsid protein	Phi19.3_06
639	Major capsid protein	Phi19_06
640	Major capsid protein	Phi4.2_07
641	Major capsid protein	Phi40_08
642	Major capsid protein	Phi43_06
643	Major capsid protein	Phi44_07
644	Major capsid protein	Phi4_06
645	Major capsid protein	Phi5.12_07
646	Major capsid protein	Phi91127_08
647	Major capsid protein	Phi93_09
648	Major capsid protein	PhiA1127_07
649	Major capsid protein	PhiA16_06
650	Major capsid protein	PhiB1127_06
651	Major capsid protein	PhiC0139_06
652	Major capsid protein	PhiD.18_06
653	Major capsid protein	PhiE1127_07
654	Major capsid protein	PhiF.17_07
655	Major capsid protein	PhiF0139_07
656	Major capsid protein	PhiG_07
657	Major capsid protein	PhiJF1_07
658	Major capsid protein	PhiL.18_08
659	Major capsid protein	PhiL.6_08
660	Major capsid protein	PhiLj_06
661	Major capsid protein	PhiM.16_08
662	Major capsid protein	PhiM.5_08
663	Major capsid protein	PhiM1127_09
664	Major capsid protein	PhiS0139_06
665	Major capsid protein	SL4_0007
666	Major capsid protein	ViridisJM2_gp06
667	Major capsid protein	bIBB29_gp06
668	Major capsid protein	bIL170p06
669	Major capsid protein	bIL170p07
670	Major capsid protein	fd13_006
671	Major capsid protein	jj50_ORF6
672	Major capsid protein	jm1_0006
673	Major capsid protein	p2_0006
674	Major capsid protein	phi7_06
675	Major capsid protein	sk1p06
676	Hypothetical protein	340_008
677	Hypothetical protein	645_007
678	Hypothetical protein	936_007
679	Hypothetical protein	ASCC191_0008
680	Hypothetical protein	ASCC273_0008
681	Hypothetical protein	ASCC281_0009

682	Hypothetical protein	ASCC284_0009
683	Hypothetical protein	ASCC287_0008
684	Hypothetical protein	ASCC310_0009
685	Hypothetical protein	ASCC324_0008
686	Hypothetical protein	ASCC337_0008
687	Hypothetical protein	ASCC356_0009
688	Hypothetical protein	ASCC358_0009
689	Hypothetical protein	ASCC365_0009
690	Hypothetical protein	ASCC368_0008
691	Hypothetical protein	ASCC395_0008
692	Hypothetical protein	ASCC397_0008
693	Hypothetical protein	ASCC406_0008
694	Hypothetical protein	ASCC454_0008
695	Hypothetical protein	ASCC460_0008
696	Hypothetical protein	ASCC465_0009
697	Hypothetical protein	ASCC473_0009
698	Hypothetical protein	ASCC476_0008
699	Hypothetical protein	ASCC489_0009
700	Hypothetical protein	ASCC497_0009
701	Hypothetical protein	ASCC502_0008
702	Hypothetical protein	ASCC506_0008
703	Hypothetical protein	ASCC527_0008
704	Hypothetical protein	ASCC531_0009
705	Hypothetical protein	ASCC532_0009
706	Hypothetical protein	ASCC544_0008
707	Hypothetical protein	CB13_0008
708	Hypothetical protein	CB14_0008
709	Hypothetical protein	CB19_0008
710	Hypothetical protein	CB20_0008
711	Hypothetical protein	LPPV008_gp08
712	Hypothetical protein	LPV712_gp007
713	Hypothetical protein	P113G_007
714	Hypothetical protein	P272_007
715	Hypothetical protein	P475_007
716	Hypothetical protein	P680_07
717	Hypothetical protein	PastusJM3_07
718	Hypothetical protein	Phi10.5_08
719	Hypothetical protein	Phi109_09
720	Hypothetical protein	Phi114_08
721	Hypothetical protein	Phi129_07
722	Hypothetical protein	Phi1316_07
723	Hypothetical protein	Phi145_08
724	Hypothetical protein	Phi155_09
725	Hypothetical protein	Phi15_08
726	Hypothetical protein	Phi16_09
727	Hypothetical protein	Phi17_09
728	Hypothetical protein	Phi19.2_07
729	Hypothetical protein	Phi19.3_07
730	Hypothetical protein	Phi19_07
731	Hypothetical protein	Phi4.2_08

732	Hypothetical protein	Phi40_09
733	Hypothetical protein	Phi43_07
734	Hypothetical protein	Phi44_08
735	Hypothetical protein	Phi4_07
736	Hypothetical protein	Phi5.12_08
737	Hypothetical protein	Phi91127_09
738	Hypothetical protein	Phi93_10
739	Hypothetical protein	PhiA1127_08
740	Hypothetical protein	PhiA16_07
741	Hypothetical protein	PhiB1127_07
742	Hypothetical protein	PhiC0139_07
743	Hypothetical protein	PhiD.18_07
744	Hypothetical protein	PhiE1127_08
745	Hypothetical protein	PhiF.17_08
746	Hypothetical protein	PhiF0139_08
747	Hypothetical protein	PhiG_08
748	Hypothetical protein	PhiJF1_08
749	Hypothetical protein	PhiL.18_09
750	Hypothetical protein	PhiL.6_09
751	Hypothetical protein	PhiLj_07
752	Hypothetical protein	PhiM.16_09
753	Hypothetical protein	PhiM.5_09
754	Hypothetical protein	PhiM1127_10
755	Hypothetical protein	PhiS0139_07
756	Hypothetical protein	SL4_0008
757	Hypothetical protein	ViridisJM2_gp07
758	Hypothetical protein	bIBB29_gp07
759	Hypothetical protein	bIL170p08
760	Hypothetical protein	fd13_007
761	Hypothetical protein	jj50_ORF7
762	Hypothetical protein	jm1_0007
763	Hypothetical protein	p2_0007
764	Hypothetical protein	phi7_07
765	Hypothetical protein	sk1p07
766	Connector	340_009
767	Connector	645_008
768	Connector	936_008
769	Connector	ASCC191_0009
770	Connector	ASCC273_0009
771	Connector	ASCC281_0010
772	Connector	ASCC284_0010
773	Connector	ASCC287_0009
774	Connector	ASCC310_0010
775	Connector	ASCC324_0009
776	Connector	ASCC337_0009
777	Connector	ASCC356_0010
778	Connector	ASCC358_0010
779	Connector	ASCC365_0010
780	Connector	ASCC368_0009
781	Connector	ASCC395_0009

782	Connector	ASCC397_0009
783	Connector	ASCC406_0009
784	Connector	ASCC454_0009
785	Connector	ASCC460_0009
786	Connector	ASCC465_0010
787	Connector	ASCC473_0010
788	Connector	ASCC476_0009
789	Connector	ASCC489_0010
790	Connector	ASCC497_0010
791	Connector	ASCC502_0009
792	Connector	ASCC506_0009
793	Connector	ASCC527_0009
794	Connector	ASCC531_0010
795	Connector	ASCC532_0010
796	Connector	ASCC544_0009
797	Connector	CB13_0009
798	Connector	CB14_0009
799	Connector	CB19_0009
800	Connector	CB20_0009
801	Connector	LPPV008_gp09
802	Connector	LPV712_gp008
803	Connector	P113G_008
804	Connector	P272_008
805	Connector	P475_008
806	Connector	P680_08
807	Connector	PastusJM3_08
808	Connector	Phi10.5_09
809	Connector	Phi109_10
810	Connector	Phi114_09
811	Connector	Phi129_08
812	Connector	Phi1316_08
813	Connector	Phi145_09
814	Connector	Phi155_10
815	Connector	Phi15_09
816	Connector	Phi16_10
817	Connector	Phi17_10
818	Connector	Phi19.2_08
819	Connector	Phi19.3_08
820	Connector	Phi19_08
821	Connector	Phi4.2_09
822	Connector	Phi40_10
823	Connector	Phi43_08
824	Connector	Phi44_09
825	Connector	Phi4_08
826	Connector	Phi5.12_09
827	Connector	Phi91127_10
828	Connector	Phi93_11
829	Connector	PhiA1127_09
830	Connector	PhiA16_08
831	Connector	PhiB1127_08

832	Connector	PhiC0139_08
833	Connector	PhiD.18_08
834	Connector	PhiE1127_09
835	Connector	PhiF.17_09
836	Connector	PhiF0139_09
837	Connector	PhiG_09
838	Connector	PhiJF1_09
839	Connector	PhiL.18_10
840	Connector	PhiL.6_10
841	Connector	PhiLj_08
842	Connector	PhiM.16_10
843	Connector	PhiM.5_10
844	Connector	PhiM1127_11
845	Connector	PhiS0139_08
846	Connector	SL4_0009
847	Connector	ViridisJM2_gp08
848	Connector	bIBB29_gp08
849	Connector	bIL170p09
850	Connector	fd13_008
851	Connector	jj50_ORF8
852	Connector	jm1_0008
853	Connector	p2_0008
854	Connector	phi7_08
855	Connector	sk1p08
856	Stopper	340_0010
857	Stopper	645_009
858	Stopper	936_009
859	Stopper	ASCC191_0010
860	Stopper	ASCC191_0013
861	Stopper	ASCC273_0010
862	Stopper	ASCC273_0013
863	Stopper	ASCC281_0011
864	Stopper	ASCC284_0011
865	Stopper	ASCC287_0010
866	Stopper	ASCC287_0013
867	Stopper	ASCC310_0011
868	Stopper	ASCC324_0010
869	Stopper	ASCC324_0013
870	Stopper	ASCC337_0010
871	Stopper	ASCC337_0013
872	Stopper	ASCC356_0011
873	Stopper	ASCC358_0011
874	Stopper	ASCC365_0011
875	Stopper	ASCC368_0010
876	Stopper	ASCC368_0013
877	Stopper	ASCC395_0010
878	Stopper	ASCC395_0013
879	Stopper	ASCC397_0010
880	Stopper	ASCC397_0013
881	Stopper	ASCC406_0010

882	Stopper	ASCC406_0013
883	Stopper	ASCC454_0010
884	Stopper	ASCC454_0013
885	Stopper	ASCC460_0010
886	Stopper	ASCC460_0013
887	Stopper	ASCC465_0011
888	Stopper	ASCC473_0011
889	Stopper	ASCC476_0010
890	Stopper	ASCC476_0013
891	Stopper	ASCC489_0011
892	Stopper	ASCC497_0011
893	Stopper	ASCC502_0010
894	Stopper	ASCC502_0013
895	Stopper	ASCC506_0010
896	Stopper	ASCC506_0013
897	Stopper	ASCC527_0010
898	Stopper	ASCC527_0013
899	Stopper	ASCC531_0011
900	Stopper	ASCC532_0011
901	Stopper	ASCC544_0010
902	Stopper	ASCC544_0013
903	Stopper	CB13_0010
904	Stopper	CB14_0010
905	Stopper	CB19_0010
906	Stopper	CB20_0010
907	Stopper	LPPV008_gp10
908	Stopper	LPV712_gp009
909	Stopper	P113G_009
910	Stopper	P272_009
911	Stopper	P475_0010
912	Stopper	P680_09
913	Stopper	PastusJM3_09
914	Stopper	Phi10.5_11
915	Stopper	Phi109_12
916	Stopper	Phi114_10
917	Stopper	Phi129_09
918	Stopper	Phi1316_09
919	Stopper	Phi145_11
920	Stopper	Phi155_11
921	Stopper	Phi15_10
922	Stopper	Phi16_12
923	Stopper	Phi17_11
924	Stopper	Phi19.2_09
925	Stopper	Phi19.3_09
926	Stopper	Phi19_09
927	Stopper	Phi4.2_10
928	Stopper	Phi40_12
929	Stopper	Phi43_09
930	Stopper	Phi44_10
931	Stopper	Phi4_10

932	Stopper	Phi5.12_10
933	Stopper	Phi91127_11
934	Stopper	Phi93_13
935	Stopper	PhiA1127_10
936	Stopper	PhiA16_09
937	Stopper	PhiB1127_09
938	Stopper	PhiC0139_09
939	Stopper	PhiD.18_09
940	Stopper	PhiE1127_10
941	Stopper	PhiF.17_10
942	Stopper	PhiF0139_10
943	Stopper	PhiG_10
944	Stopper	PhiJF1_11
945	Stopper	PhiL.18_11
946	Stopper	PhiL.6_11
947	Stopper	PhiLj_09
948	Stopper	PhiM.16_11
949	Stopper	PhiM.5_11
950	Stopper	PhiM1127_12
951	Stopper	PhiS0139_09
952	Stopper	SL4_0011
953	Stopper	ViridisJM2_gp09
954	Stopper	bIBB29_gp09
955	Stopper	bIL170p10
956	Stopper	fd13_0010
957	Stopper	jj50_ORF9
958	Stopper	jm1_0009
959	Stopper	p2_0009
960	Stopper	phi7_09
961	Stopper	sk1p09
962	Tail terminator	340_0011
963	Tail terminator	645_0010
964	Tail terminator	936_0010
965	Tail terminator	ASCC191_0011
966	Tail terminator	ASCC273_0011
967	Tail terminator	ASCC281_0012
968	Tail terminator	ASCC284_0012
969	Tail terminator	ASCC287_0011
970	Tail terminator	ASCC310_0012
971	Tail terminator	ASCC324_0011
972	Tail terminator	ASCC337_0011
973	Tail terminator	ASCC356_0012
974	Tail terminator	ASCC358_0012
975	Tail terminator	ASCC365_0012
976	Tail terminator	ASCC368_0011
977	Tail terminator	ASCC395_0011
978	Tail terminator	ASCC397_0011
979	Tail terminator	ASCC406_0011
980	Tail terminator	ASCC454_0011
981	Tail terminator	ASCC460_0011

982	Tail terminator	ASCC465_0012
983	Tail terminator	ASCC473_0012
984	Tail terminator	ASCC476_0011
985	Tail terminator	ASCC489_0012
986	Tail terminator	ASCC497_0012
987	Tail terminator	ASCC502_0011
988	Tail terminator	ASCC506_0011
989	Tail terminator	ASCC527_0011
990	Tail terminator	ASCC531_0012
991	Tail terminator	ASCC532_0012
992	Tail terminator	ASCC544_0011
993	Tail terminator	CB13_0011
994	Tail terminator	CB14_0011
995	Tail terminator	CB19_0011
996	Tail terminator	CB20_0011
997	Tail terminator	LPPV008_gp11
998	Tail terminator	LPV712_gp010
999	Tail terminator	P113G_0010
1000	Tail terminator	P272_0010
1001	Tail terminator	P475_0011
1002	Tail terminator	P680_10
1003	Tail terminator	PastusJM3_10
1004	Tail terminator	Phi10.5_12
1005	Tail terminator	Phi109_13
1006	Tail terminator	Phi114_11
1007	Tail terminator	Phi129_10
1008	Tail terminator	Phi1316_10
1009	Tail terminator	Phi145_12
1010	Tail terminator	Phi155_12
1011	Tail terminator	Phi15_11
1012	Tail terminator	Phi16_13
1013	Tail terminator	Phi17_12
1014	Tail terminator	Phi19.2_10
1015	Tail terminator	Phi19.3_10
1016	Tail terminator	Phi19_10
1017	Tail terminator	Phi4.2_11
1018	Tail terminator	Phi40_13
1019	Tail terminator	Phi43_10
1020	Tail terminator	Phi44_11
1021	Tail terminator	Phi4_11
1022	Tail terminator	Phi5.12_11
1023	Tail terminator	Phi91127_12
1024	Tail terminator	Phi93_14
1025	Tail terminator	PhiA1127_11
1026	Tail terminator	PhiA16_10
1027	Tail terminator	PhiB1127_10
1028	Tail terminator	PhiC0139_10
1029	Tail terminator	PhiD.18_10
1030	Tail terminator	PhiE1127_11
1031	Tail terminator	PhiF.17_11

1032	Tail terminator	PhiF0139_11
1033	Tail terminator	PhiG_11
1034	Tail terminator	PhiJF1_12
1035	Tail terminator	PhiL.18_12
1036	Tail terminator	PhiL.6_12
1037	Tail terminator	PhiLj_10
1038	Tail terminator	PhiM.16_12
1039	Tail terminator	PhiM.5_12
1040	Tail terminator	PhiM1127_13
1041	Tail terminator	PhiS0139_10
1042	Tail terminator	SL4_0012
1043	Tail terminator	ViridisJM2_gp10
1044	Tail terminator	bIBB29_gp10
1045	Tail terminator	bIL170p11
1046	Tail terminator	fd13_0011
1047	Tail terminator	jj50_ORF10
1048	Tail terminator	jm1_0010
1049	Tail terminator	p2_0010
1050	Tail terminator	phi7_10
1051	Tail terminator	sk1p10
1052	Major tail protein	340_0013
1053	Major tail protein	645_0012
1054	Major tail protein	936_0011
1055	Major tail protein	ASCC191_0014
1056	Major tail protein	ASCC273_0014
1057	Major tail protein	ASCC281_0014
1058	Major tail protein	ASCC284_0014
1059	Major tail protein	ASCC287_0014
1060	Major tail protein	ASCC310_0014
1061	Major tail protein	ASCC324_0014
1062	Major tail protein	ASCC337_0014
1063	Major tail protein	ASCC356_0014
1064	Major tail protein	ASCC358_0014
1065	Major tail protein	ASCC365_0014
1066	Major tail protein	ASCC368_0014
1067	Major tail protein	ASCC395_0014
1068	Major tail protein	ASCC397_0014
1069	Major tail protein	ASCC406_0014
1070	Major tail protein	ASCC454_0014
1071	Major tail protein	ASCC460_0014
1072	Major tail protein	ASCC465_0014
1073	Major tail protein	ASCC473_0014
1074	Major tail protein	ASCC476_0014
1075	Major tail protein	ASCC489_0014
1076	Major tail protein	ASCC497_0014
1077	Major tail protein	ASCC502_0014
1078	Major tail protein	ASCC506_0014
1079	Major tail protein	ASCC527_0014
1080	Major tail protein	ASCC531_0014
1081	Major tail protein	ASCC532_0014

1082	Major tail protein	ASCC544_0014
1083	Major tail protein	CB13_0013
1084	Major tail protein	CB14_0013
1085	Major tail protein	CB19_0013
1086	Major tail protein	CB20_0013
1087	Major tail protein	LPPV008_gp13
1088	Major tail protein	LPV712_gp011
1089	Major tail protein	P113G_0011
1090	Major tail protein	P272_0011
1091	Major tail protein	P475_0012
1092	Major tail protein	P680_12
1093	Major tail protein	PastusJM3_12
1094	Major tail protein	Phi10.5_13
1095	Major tail protein	Phi109_14
1096	Major tail protein	Phi114_13
1097	Major tail protein	Phi129_12
1098	Major tail protein	Phi1316_12
1099	Major tail protein	Phi145_13
1100	Major tail protein	Phi155_14
1101	Major tail protein	Phi15_13
1102	Major tail protein	Phi16_14
1103	Major tail protein	Phi17_13
1104	Major tail protein	Phi19.2_12
1105	Major tail protein	Phi19.3_12
1106	Major tail protein	Phi19_12
1107	Major tail protein	Phi4.2_12
1108	Major tail protein	Phi40_14
1109	Major tail protein	Phi43_12
1110	Major tail protein	Phi44_13
1111	Major tail protein	Phi4_13
1112	Major tail protein	Phi5.12_12
1113	Major tail protein	Phi91127_13
1114	Major tail protein	Phi93_15
1115	Major tail protein	PhiA1127_13
1116	Major tail protein	PhiA16_12
1117	Major tail protein	PhiB1127_12
1118	Major tail protein	PhiC0139_12
1119	Major tail protein	PhiD.18_12
1120	Major tail protein	PhiE1127_12
1121	Major tail protein	PhiF.17_13
1122	Major tail protein	PhiF0139_13
1123	Major tail protein	PhiG_13
1124	Major tail protein	PhiJF1_14
1125	Major tail protein	PhiL.18_13
1126	Major tail protein	PhiL.6_13
1127	Major tail protein	PhiLj_12
1128	Major tail protein	PhiM.16_14
1129	Major tail protein	PhiM.5_13
1130	Major tail protein	PhiM1127_14
1131	Major tail protein	PhiS0139_12

1132	Major tail protein	SL4_0013
1133	Major tail protein	ViridisJM2_gp12
1134	Major tail protein	bIBB29_gp11
1135	Major tail protein	bIL170p13
1136	Major tail protein	fd13_0013
1137	Major tail protein	jj50_ORF11
1138	Major tail protein	jm1_0012
1139	Major tail protein	p2_0011
1140	Major tail protein	phi7_12
1141	Major tail protein	sk1p11
1142	Chaperone Protein	340_0014
1143	Chaperone Protein	645_0013
1144	Chaperone Protein	936_0012
1145	Chaperone Protein	ASCC191_0015
1146	Chaperone Protein	ASCC273_0015
1147	Chaperone Protein	ASCC281_0015
1148	Chaperone Protein	ASCC284_0015
1149	Chaperone Protein	ASCC287_0015
1150	Chaperone Protein	ASCC310_0015
1151	Chaperone Protein	ASCC324_0015
1152	Chaperone Protein	ASCC337_0015
1153	Chaperone Protein	ASCC356_0015
1154	Chaperone Protein	ASCC358_0015
1155	Chaperone Protein	ASCC365_0015
1156	Chaperone Protein	ASCC368_0015
1157	Chaperone Protein	ASCC395_0015
1158	Chaperone Protein	ASCC397_0015
1159	Chaperone Protein	ASCC406_0015
1160	Chaperone Protein	ASCC454_0015
1161	Chaperone Protein	ASCC460_0015
1162	Chaperone Protein	ASCC465_0015
1163	Chaperone Protein	ASCC473_0015
1164	Chaperone Protein	ASCC476_0015
1165	Chaperone Protein	ASCC489_0015
1166	Chaperone Protein	ASCC497_0015
1167	Chaperone Protein	ASCC502_0015
1168	Chaperone Protein	ASCC506_0015
1169	Chaperone Protein	ASCC527_0015
1170	Chaperone Protein	ASCC531_0015
1171	Chaperone Protein	ASCC532_0015
1172	Chaperone Protein	ASCC544_0015
1173	Chaperone Protein	CB13_0014
1174	Chaperone Protein	CB14_0014
1175	Chaperone Protein	CB19_0014
1176	Chaperone Protein	CB20_0014
1177	Chaperone Protein	LPPV008_gp14
1178	Chaperone Protein	LPV712_gp013
1179	Chaperone Protein	P113G_0013
1180	Chaperone Protein	P272_0013
1181	Chaperone Protein	P475_0014

1182	Chaperone Protein	P680_13
1183	Chaperone Protein	PastusJM3_13
1184	Chaperone Protein	Phi10.5_14
1185	Chaperone Protein	Phi109_16
1186	Chaperone Protein	Phi114_14
1187	Chaperone Protein	Phi129_13
1188	Chaperone Protein	Phi1316_13
1189	Chaperone Protein	Phi145_15
1190	Chaperone Protein	Phi155_15
1191	Chaperone Protein	Phi15_14
1192	Chaperone Protein	Phi16_16
1193	Chaperone Protein	Phi17_15
1194	Chaperone Protein	Phi19.2_13
1195	Chaperone Protein	Phi19.3_13
1196	Chaperone Protein	Phi19_13
1197	Chaperone Protein	Phi4.2_14
1198	Chaperone Protein	Phi40_16
1199	Chaperone Protein	Phi43_13
1200	Chaperone Protein	Phi44_14
1201	Chaperone Protein	Phi4_14
1202	Chaperone Protein	Phi5.12_14
1203	Chaperone Protein	Phi91127_15
1204	Chaperone Protein	Phi93_17
1205	Chaperone Protein	PhiA1127_14
1206	Chaperone Protein	PhiA16_13
1207	Chaperone Protein	PhiB1127_14
1208	Chaperone Protein	PhiC0139_14
1209	Chaperone Protein	PhiD.18_13
1210	Chaperone Protein	PhiE1127_14
1211	Chaperone Protein	PhiF.17_14
1212	Chaperone Protein	PhiF0139_14
1213	Chaperone Protein	PhiG_14
1214	Chaperone Protein	PhiJF1_15
1215	Chaperone Protein	PhiL.18_15
1216	Chaperone Protein	PhiL.6_15
1217	Chaperone Protein	PhiLj_13
1218	Chaperone Protein	PhiM.16_15
1219	Chaperone Protein	PhiM.5_15
1220	Chaperone Protein	PhiM1127_16
1221	Chaperone Protein	PhiS0139_13
1222	Chaperone Protein	SL4_0014
1223	Chaperone Protein	ViridisJM2_gp13
1224	Chaperone Protein	bIBB29_gp13
1225	Chaperone Protein	bIL170p14
1226	Chaperone Protein	fd13_0014
1227	Chaperone Protein	jj50_ORF12
1228	Chaperone Protein	jm1_0013
1229	Chaperone Protein	p2_0012
1230	Chaperone Protein	phi7_14
1231	Chaperone Protein	sk1p12

1232	Chaperone Protein	340_0015
1233	Chaperone Protein	645_0014
1234	Chaperone Protein	936_0013
1235	Chaperone Protein	ASCC191_0016
1236	Chaperone Protein	ASCC273_0016
1237	Chaperone Protein	ASCC281_0016
1238	Chaperone Protein	ASCC284_0016
1239	Chaperone Protein	ASCC287_0016
1240	Chaperone Protein	ASCC310_0016
1241	Chaperone Protein	ASCC324_0016
1242	Chaperone Protein	ASCC337_0016
1243	Chaperone Protein	ASCC356_0016
1244	Chaperone Protein	ASCC358_0016
1245	Chaperone Protein	ASCC365_0016
1246	Chaperone Protein	ASCC368_0016
1247	Chaperone Protein	ASCC395_0016
1248	Chaperone Protein	ASCC397_0016
1249	Chaperone Protein	ASCC406_0016
1250	Chaperone Protein	ASCC454_0016
1251	Chaperone Protein	ASCC460_0016
1252	Chaperone Protein	ASCC465_0016
1253	Chaperone Protein	ASCC473_0016
1254	Chaperone Protein	ASCC476_0016
1255	Chaperone Protein	ASCC489_0016
1256	Chaperone Protein	ASCC497_0016
1257	Chaperone Protein	ASCC502_0016
1258	Chaperone Protein	ASCC506_0016
1259	Chaperone Protein	ASCC527_0016
1260	Chaperone Protein	ASCC531_0016
1261	Chaperone Protein	ASCC532_0016
1262	Chaperone Protein	ASCC544_0016
1263	Chaperone Protein	CB13_0015
1264	Chaperone Protein	CB14_0015
1265	Chaperone Protein	CB19_0015
1266	Chaperone Protein	CB20_0015
1267	Chaperone Protein	LPPV008_gp15
1268	Chaperone Protein	LPV712_gp014
1269	Chaperone Protein	P113G_0014
1270	Chaperone Protein	P272_0014
1271	Chaperone Protein	P475_0015
1272	Chaperone Protein	P680_14
1273	Chaperone Protein	PastusJM3_14
1274	Chaperone Protein	Phi10.5_15
1275	Chaperone Protein	Phi109_17
1276	Chaperone Protein	Phi114_15
1277	Chaperone Protein	Phi129_14
1278	Chaperone Protein	Phi1316_14
1279	Chaperone Protein	Phi145_16
1280	Chaperone Protein	Phi155_16
1281	Chaperone Protein	Phi15_15

1282	Chaperone Protein	Phi16_17
1283	Chaperone Protein	Phi17_16
1284	Chaperone Protein	Phi19.2_14
1285	Chaperone Protein	Phi19.3_14
1286	Chaperone Protein	Phi19_14
1287	Chaperone Protein	Phi4.2_15
1288	Chaperone Protein	Phi40_17
1289	Chaperone Protein	Phi43_14
1290	Chaperone Protein	Phi44_15
1291	Chaperone Protein	Phi4_15
1292	Chaperone Protein	Phi5.12_15
1293	Chaperone Protein	Phi91127_16
1294	Chaperone Protein	Phi93_18
1295	Chaperone Protein	PhiA1127_15
1296	Chaperone Protein	PhiA16_14
1297	Chaperone Protein	PhiB1127_15
1298	Chaperone Protein	PhiC0139_15
1299	Chaperone Protein	PhiD.18_14
1300	Chaperone Protein	PhiE1127_15
1301	Chaperone Protein	PhiF.17_15
1302	Chaperone Protein	PhiF0139_15
1303	Chaperone Protein	PhiG_15
1304	Chaperone Protein	PhiJF1_16
1305	Chaperone Protein	PhiL.18_16
1306	Chaperone Protein	PhiL.6_16
1307	Chaperone Protein	PhiLj_14
1308	Chaperone Protein	PhiM.16_16
1309	Chaperone Protein	PhiM.5_16
1310	Chaperone Protein	PhiM1127_17
1311	Chaperone Protein	PhiS0139_14
1312	Chaperone Protein	SL4_0015
1313	Chaperone Protein	ViridisJM2_gp14
1314	Chaperone Protein	bIBB29_gp14
1315	Chaperone Protein	bIL170p15
1316	Chaperone Protein	fd13_0015
1317	Chaperone Protein	jj50_ORF13
1318	Chaperone Protein	jm1_0014
1319	Chaperone Protein	p2_0013
1320	Chaperone Protein	phi7_15
1321	Chaperone Protein	sk1p13
1322	Tape Measure protein	340_0016
1323	Tape Measure protein	645_0015
1324	Tape Measure protein	936_0014
1325	Tape Measure protein	ASCC191_0017
1326	Tape Measure protein	ASCC273_0017
1327	Tape Measure protein	ASCC281_0017
1328	Tape Measure protein	ASCC284_0017
1329	Tape Measure protein	ASCC287_0017
1330	Tape Measure protein	ASCC310_0017
1331	Tape Measure protein	ASCC324_0017

1332 Tape Measure protein ASCC337_0017
1333 Tape Measure protein ASCC356_0017
1334 Tape Measure protein ASCC358_0017
1335 Tape Measure protein ASCC365_0017
1336 Tape Measure protein ASCC368_0017
1337 Tape Measure protein ASCC395_0017
1338 Tape Measure protein ASCC397_0017
1339 Tape Measure protein ASCC406_0017
1340 Tape Measure protein ASCC454_0017
1341 Tape Measure protein ASCC460_0017
1342 Tape Measure protein ASCC465_0017
1343 Tape Measure protein ASCC473_0017
1344 Tape Measure protein ASCC476_0017
1345 Tape Measure protein ASCC489_0017
1346 Tape Measure protein ASCC497_0017
1347 Tape Measure protein ASCC502_0017
1348 Tape Measure protein ASCC506_0017
1349 Tape Measure protein ASCC527_0017
1350 Tape Measure protein ASCC531_0017
1351 Tape Measure protein ASCC532_0017
1352 Tape Measure protein ASCC544_0017
1353 Tape Measure protein CB13_0016
1354 Tape Measure protein CB14_0016
1355 Tape Measure protein CB19_0016
1356 Tape Measure protein CB20_0016
1357 Tape Measure protein LPPV008_gp16
1358 Tape Measure protein LPV712_gp015
1359 Tape Measure protein P113G_0015
1360 Tape Measure protein P272_0015
1361 Tape Measure protein P475_0016
1362 Tape Measure protein P680_15
1363 Tape Measure protein PastusJM3_15
1364 Tape Measure protein Phi10.5_16
1365 Tape Measure protein Phi109_18
1366 Tape Measure protein Phi114_16
1367 Tape Measure protein Phi129_15
1368 Tape Measure protein Phi1316_15
1369 Tape Measure protein Phi145_17
1370 Tape Measure protein Phi155_17
1371 Tape Measure protein Phi15_16
1372 Tape Measure protein Phi16_18
1373 Tape Measure protein Phi17_17
1374 Tape Measure protein Phi19.2_15
1375 Tape Measure protein Phi19.3_15
1376 Tape Measure protein Phi19_15
1377 Tape Measure protein Phi4.2_16
1378 Tape Measure protein Phi40_18
1379 Tape Measure protein Phi43_15
1380 Tape Measure protein Phi44_16
1381 Tape Measure protein Phi4_16

1382	Tape Measure protein	Phi5.12_16
1383	Tape Measure protein	Phi91127_17
1384	Tape Measure protein	Phi93_19
1385	Tape Measure protein	PhiA1127_16
1386	Tape Measure protein	PhiA16_15
1387	Tape Measure protein	PhiB1127_16
1388	Tape Measure protein	PhiC0139_16
1389	Tape Measure protein	PhiD.18_15
1390	Tape Measure protein	PhiE1127_16
1391	Tape Measure protein	PhiF.17_16
1392	Tape Measure protein	PhiF0139_16
1393	Tape Measure protein	PhiG_16
1394	Tape Measure protein	PhiJF1_17
1395	Tape Measure protein	PhiL.18_17
1396	Tape Measure protein	PhiL.6_17
1397	Tape Measure protein	PhiLj_15
1398	Tape Measure protein	PhiM.16_17
1399	Tape Measure protein	PhiM.5_17
1400	Tape Measure protein	PhiM1127_18
1401	Tape Measure protein	PhiS0139_15
1402	Tape Measure protein	SL4_0016
1403	Tape Measure protein	ViridisJM2_gp15
1404	Tape Measure protein	bIBB29_gp15
1405	Tape Measure protein	bIL170p16
1406	Tape Measure protein	fd13_0016
1407	Tape Measure protein	jj50_ORF14
1408	Tape Measure protein	jm1_0015
1409	Tape Measure protein	p2_0014
1410	Tape Measure protein	phi7_16
1411	Tape Measure protein	sk1p14
1412	Distal tail protein	340_0017
1413	Distal tail protein	645_0016
1414	Distal tail protein	936_0015
1415	Distal tail protein	ASCC191_0018
1416	Distal tail protein	ASCC273_0018
1417	Distal tail protein	ASCC281_0018
1418	Distal tail protein	ASCC284_0018
1419	Distal tail protein	ASCC287_0018
1420	Distal tail protein	ASCC310_0018
1421	Distal tail protein	ASCC324_0018
1422	Distal tail protein	ASCC337_0018
1423	Distal tail protein	ASCC356_0018
1424	Distal tail protein	ASCC358_0018
1425	Distal tail protein	ASCC365_0018
1426	Distal tail protein	ASCC368_0018
1427	Distal tail protein	ASCC395_0018
1428	Distal tail protein	ASCC397_0018
1429	Distal tail protein	ASCC406_0018
1430	Distal tail protein	ASCC454_0018
1431	Distal tail protein	ASCC460_0018

1432	Distal tail protein	ASCC465_0018
1433	Distal tail protein	ASCC473_0018
1434	Distal tail protein	ASCC476_0018
1435	Distal tail protein	ASCC489_0018
1436	Distal tail protein	ASCC497_0018
1437	Distal tail protein	ASCC502_0018
1438	Distal tail protein	ASCC506_0018
1439	Distal tail protein	ASCC527_0018
1440	Distal tail protein	ASCC531_0018
1441	Distal tail protein	ASCC532_0018
1442	Distal tail protein	ASCC544_0018
1443	Distal tail protein	CB13_0017
1444	Distal tail protein	CB14_0017
1445	Distal tail protein	CB19_0017
1446	Distal tail protein	CB20_0017
1447	Distal tail protein	LPPV008_gp17
1448	Distal tail protein	LPV712_gp016
1449	Distal tail protein	P113G_0016
1450	Distal tail protein	P272_0016
1451	Distal tail protein	P475_0017
1452	Distal tail protein	P680_16
1453	Distal tail protein	PastusJM3_16
1454	Distal tail protein	Phi10.5_17
1455	Distal tail protein	Phi109_19
1456	Distal tail protein	Phi114_17
1457	Distal tail protein	Phi129_16
1458	Distal tail protein	Phi1316_16
1459	Distal tail protein	Phi145_18
1460	Distal tail protein	Phi155_18
1461	Distal tail protein	Phi15_17
1462	Distal tail protein	Phi16_19
1463	Distal tail protein	Phi17_18
1464	Distal tail protein	Phi19.2_16
1465	Distal tail protein	Phi19.3_16
1466	Distal tail protein	Phi19_16
1467	Distal tail protein	Phi4.2_17
1468	Distal tail protein	Phi40_19
1469	Distal tail protein	Phi43_16
1470	Distal tail protein	Phi44_17
1471	Distal tail protein	Phi4_17
1472	Distal tail protein	Phi5.12_17
1473	Distal tail protein	Phi91127_18
1474	Distal tail protein	Phi93_20
1475	Distal tail protein	PhiA1127_17
1476	Distal tail protein	PhiA16_16
1477	Distal tail protein	PhiB1127_17
1478	Distal tail protein	PhiC0139_17
1479	Distal tail protein	PhiD.18_16
1480	Distal tail protein	PhiE1127_17
1481	Distal tail protein	PhiF.17_17

1482	Distal tail protein	PhiF0139_17
1483	Distal tail protein	PhiG_17
1484	Distal tail protein	PhiJF1_18
1485	Distal tail protein	PhiL.18_18
1486	Distal tail protein	PhiL.6_18
1487	Distal tail protein	PhiLj_16
1488	Distal tail protein	PhiM.16_18
1489	Distal tail protein	PhiM.5_18
1490	Distal tail protein	PhiM1127_19
1491	Distal tail protein	PhiS0139_16
1492	Distal tail protein	SL4_0017
1493	Distal tail protein	ViridisJM2_gp16
1494	Distal tail protein	bIBB29_gp16
1495	Distal tail protein	bIL170p17
1496	Distal tail protein	fd13_0017
1497	Distal tail protein	jj50_ORF15
1498	Distal tail protein	jm1_0016
1499	Distal tail protein	p2_0015
1500	Distal tail protein	phi7_17
1501	Distal tail protein	sk1p15
1502	Tail associated lysin	340_0018
1503	Tail associated lysin	645_0017
1504	Tail associated lysin	936_0016
1505	Tail associated lysin	ASCC191_0019
1506	Tail associated lysin	ASCC273_0019
1507	Tail associated lysin	ASCC281_0019
1508	Tail associated lysin	ASCC284_0019
1509	Tail associated lysin	ASCC287_0019
1510	Tail associated lysin	ASCC310_0019
1511	Tail associated lysin	ASCC324_0019
1512	Tail associated lysin	ASCC337_0019
1513	Tail associated lysin	ASCC356_0019
1514	Tail associated lysin	ASCC358_0019
1515	Tail associated lysin	ASCC365_0019
1516	Tail associated lysin	ASCC368_0019
1517	Tail associated lysin	ASCC395_0019
1518	Tail associated lysin	ASCC397_0019
1519	Tail associated lysin	ASCC406_0019
1520	Tail associated lysin	ASCC454_0019
1521	Tail associated lysin	ASCC460_0019
1522	Tail associated lysin	ASCC465_0019
1523	Tail associated lysin	ASCC473_0019
1524	Tail associated lysin	ASCC476_0019
1525	Tail associated lysin	ASCC489_0019
1526	Tail associated lysin	ASCC497_0019
1527	Tail associated lysin	ASCC502_0019
1528	Tail associated lysin	ASCC506_0019
1529	Tail associated lysin	ASCC527_0019
1530	Tail associated lysin	ASCC531_0019
1531	Tail associated lysin	ASCC532_0019

1532	Tail associated lysin	ASCC544_0019
1533	Tail associated lysin	CB13_0018
1534	Tail associated lysin	CB14_0018
1535	Tail associated lysin	CB19_0018
1536	Tail associated lysin	CB20_0018
1537	Tail associated lysin	LPPV008_gp18
1538	Tail associated lysin	LPV712_gp017
1539	Tail associated lysin	P113G_0017
1540	Tail associated lysin	P272_0017
1541	Tail associated lysin	P475_0018
1542	Tail associated lysin	P680_17
1543	Tail associated lysin	PastusJM3_17
1544	Tail associated lysin	Phi10.5_18
1545	Tail associated lysin	Phi109_20
1546	Tail associated lysin	Phi114_18
1547	Tail associated lysin	Phi129_17
1548	Tail associated lysin	Phi1316_17
1549	Tail associated lysin	Phi145_19
1550	Tail associated lysin	Phi155_19
1551	Tail associated lysin	Phi15_18
1552	Tail associated lysin	Phi16_20
1553	Tail associated lysin	Phi17_19
1554	Tail associated lysin	Phi19.2_17
1555	Tail associated lysin	Phi19.3_17
1556	Tail associated lysin	Phi19_17
1557	Tail associated lysin	Phi4.2_18
1558	Tail associated lysin	Phi40_20
1559	Tail associated lysin	Phi43_17
1560	Tail associated lysin	Phi44_18
1561	Tail associated lysin	Phi4_18
1562	Tail associated lysin	Phi5.12_18
1563	Tail associated lysin	Phi91127_19
1564	Tail associated lysin	Phi93_21
1565	Tail associated lysin	PhiA1127_18
1566	Tail associated lysin	PhiA16_17
1567	Tail associated lysin	PhiB1127_18
1568	Tail associated lysin	PhiC0139_18
1569	Tail associated lysin	PhiD.18_17
1570	Tail associated lysin	PhiE1127_18
1571	Tail associated lysin	PhiF.17_18
1572	Tail associated lysin	PhiF0139_18
1573	Tail associated lysin	PhiG_18
1574	Tail associated lysin	PhiJF1_19
1575	Tail associated lysin	PhiL.18_19
1576	Tail associated lysin	PhiL.6_19
1577	Tail associated lysin	PhiLj_17
1578	Tail associated lysin	PhiM.16_19
1579	Tail associated lysin	PhiM.5_19
1580	Tail associated lysin	PhiM1127_20
1581	Tail associated lysin	PhiS0139_17

1582	Tail associated lysin	SL4_0018
1583	Tail associated lysin	ViridisJM2_gp17
1584	Tail associated lysin	bIBB29_gp17
1585	Tail associated lysin	bIL170p18
1586	Tail associated lysin	fd13_0018
1587	Tail associated lysin	jj50_ORF16
1588	Tail associated lysin	jm1_0017
1589	Tail associated lysin	p2_0016
1590	Tail associated lysin	phi7_18
1591	Tail associated lysin	sk1p16
1592	Hypothetical protein	340_0019
1593	Hypothetical protein	645_0018
1594	Hypothetical protein	936_0017
1595	Hypothetical protein	ASCC191_0020
1596	Hypothetical protein	ASCC273_0020
1597	Hypothetical protein	ASCC281_0020
1598	Hypothetical protein	ASCC284_0020
1599	Hypothetical protein	ASCC287_0020
1600	Hypothetical protein	ASCC310_0020
1601	Hypothetical protein	ASCC324_0020
1602	Hypothetical protein	ASCC337_0020
1603	Hypothetical protein	ASCC356_0020
1604	Hypothetical protein	ASCC358_0020
1605	Hypothetical protein	ASCC365_0020
1606	Hypothetical protein	ASCC368_0020
1607	Hypothetical protein	ASCC395_0020
1608	Hypothetical protein	ASCC397_0020
1609	Hypothetical protein	ASCC406_0020
1610	Hypothetical protein	ASCC454_0020
1611	Hypothetical protein	ASCC460_0020
1612	Hypothetical protein	ASCC465_0020
1613	Hypothetical protein	ASCC473_0020
1614	Hypothetical protein	ASCC476_0020
1615	Hypothetical protein	ASCC489_0020
1616	Hypothetical protein	ASCC497_0020
1617	Hypothetical protein	ASCC502_0020
1618	Hypothetical protein	ASCC506_0020
1619	Hypothetical protein	ASCC527_0020
1620	Hypothetical protein	ASCC531_0020
1621	Hypothetical protein	ASCC532_0020
1622	Hypothetical protein	ASCC544_0020
1623	Hypothetical protein	CB13_0019
1624	Hypothetical protein	CB14_0019
1625	Hypothetical protein	CB19_0019
1626	Hypothetical protein	CB20_0019
1627	Hypothetical protein	LPPV008_gp19
1628	Hypothetical protein	LPV712_gp018
1629	Hypothetical protein	P113G_0018
1630	Hypothetical protein	P272_0018
1631	Hypothetical protein	P475_0019

1632	Hypothetical protein	P680_18
1633	Hypothetical protein	PastusJM3_18
1634	Hypothetical protein	Phi10.5_19
1635	Hypothetical protein	Phi109_21
1636	Hypothetical protein	Phi114_19
1637	Hypothetical protein	Phi129_18
1638	Hypothetical protein	Phi1316_18
1639	Hypothetical protein	Phi145_20
1640	Hypothetical protein	Phi155_20
1641	Hypothetical protein	Phi15_19
1642	Hypothetical protein	Phi16_21
1643	Hypothetical protein	Phi17_20
1644	Hypothetical protein	Phi19.2_18
1645	Hypothetical protein	Phi19.3_18
1646	Hypothetical protein	Phi19_18
1647	Hypothetical protein	Phi4.2_19
1648	Hypothetical protein	Phi40_21
1649	Hypothetical protein	Phi43_18
1650	Hypothetical protein	Phi44_19
1651	Hypothetical protein	Phi4_19
1652	Hypothetical protein	Phi5.12_19
1653	Hypothetical protein	Phi91127_20
1654	Hypothetical protein	Phi93_22
1655	Hypothetical protein	PhiA1127_19
1656	Hypothetical protein	PhiA16_18
1657	Hypothetical protein	PhiB1127_19
1658	Hypothetical protein	PhiC0139_19
1659	Hypothetical protein	PhiD.18_18
1660	Hypothetical protein	PhiE1127_19
1661	Hypothetical protein	PhiF.17_19
1662	Hypothetical protein	PhiF0139_19
1663	Hypothetical protein	PhiG_19
1664	Hypothetical protein	PhiJF1_20
1665	Hypothetical protein	PhiL.18_20
1666	Hypothetical protein	PhiL.6_20
1667	Hypothetical protein	PhiLj_18
1668	Hypothetical protein	PhiM.16_20
1669	Hypothetical protein	PhiM.5_20
1670	Hypothetical protein	PhiM1127_21
1671	Hypothetical protein	PhiS0139_18
1672	Hypothetical protein	SL4_0019
1673	Hypothetical protein	ViridisJM2_gp18
1674	Hypothetical protein	bIBB29_gp18
1675	Hypothetical protein	bIL170p19
1676	Hypothetical protein	fd13_0019
1677	Hypothetical protein	jj50_ORF17
1678	Hypothetical protein	jm1_0018
1679	Hypothetical protein	p2_0017
1680	Hypothetical protein	phi7_19
1681	Hypothetical protein	sk1p17

1682	Receptor binding protein	340_0020
1683	Receptor binding protein	645_0019
1684	Receptor binding protein	936_0018
1685	Receptor binding protein	ASCC191_0021
1686	Receptor binding protein	ASCC273_0021
1687	Receptor binding protein	ASCC281_0021
1688	Receptor binding protein	ASCC284_0021
1689	Receptor binding protein	ASCC287_0021
1690	Receptor binding protein	ASCC310_0021
1691	Receptor binding protein	ASCC324_0021
1692	Receptor binding protein	ASCC337_0021
1693	Receptor binding protein	ASCC356_0021
1694	Receptor binding protein	ASCC358_0021
1695	Receptor binding protein	ASCC365_0021
1696	Receptor binding protein	ASCC368_0021
1697	Receptor binding protein	ASCC395_0021
1698	Receptor binding protein	ASCC397_0021
1699	Receptor binding protein	ASCC406_0021
1700	Receptor binding protein	ASCC454_0021
1701	Receptor binding protein	ASCC460_0021
1702	Receptor binding protein	ASCC465_0021
1703	Receptor binding protein	ASCC473_0021
1704	Receptor binding protein	ASCC476_0021
1705	Receptor binding protein	ASCC489_0021
1706	Receptor binding protein	ASCC497_0021
1707	Receptor binding protein	ASCC502_0021
1708	Receptor binding protein	ASCC506_0021
1709	Receptor binding protein	ASCC527_0021
1710	Receptor binding protein	ASCC531_0021
1711	Receptor binding protein	ASCC532_0021
1712	Receptor binding protein	ASCC544_0021
1713	Receptor binding protein	CB13_0020
1714	Receptor binding protein	CB14_0020
1715	Receptor binding protein	CB19_0020
1716	Receptor binding protein	CB20_0020
1717	Receptor binding protein	LPPV008_gp20
1718	Receptor binding protein	LPV712_gp019
1719	Receptor binding protein	P113G_0019
1720	Receptor binding protein	P272_0019
1721	Receptor binding protein	P475_0020
1722	Receptor binding protein	P680_19
1723	Receptor binding protein	PastusJM3_19
1724	Receptor binding protein	Phi10.5_20
1725	Receptor binding protein	Phi109_22
1726	Receptor binding protein	Phi114_20
1727	Receptor binding protein	Phi129_19
1728	Receptor binding protein	Phi1316_19
1729	Receptor binding protein	Phi145_21
1730	Receptor binding protein	Phi155_21
1731	Receptor binding protein	Phi15_20

1732	Receptor binding protein	Phi16_22
1733	Receptor binding protein	Phi17_21
1734	Receptor binding protein	Phi19.2_19
1735	Receptor binding protein	Phi19.3_19
1736	Receptor binding protein	Phi19_19
1737	Receptor binding protein	Phi4.2_20
1738	Receptor binding protein	Phi4.2_21
1739	Receptor binding protein	Phi40_22
1740	Receptor binding protein	Phi43_19
1741	Receptor binding protein	Phi44_20
1742	Receptor binding protein	Phi4_20
1743	Receptor binding protein	Phi5.12_20
1744	Receptor binding protein	Phi91127_21
1745	Receptor binding protein	Phi93_23
1746	Receptor binding protein	PhiA1127_20
1747	Receptor binding protein	PhiA16_19
1748	Receptor binding protein	PhiB1127_20
1749	Receptor binding protein	PhiC0139_20
1750	Receptor binding protein	PhiD.18_19
1751	Receptor binding protein	PhiE1127_20
1752	Receptor binding protein	PhiF.17_20
1753	Receptor binding protein	PhiF0139_20
1754	Receptor binding protein	PhiG_20
1755	Receptor binding protein	PhiJF1_21
1756	Receptor binding protein	PhiL.18_21
1757	Receptor binding protein	PhiL.6_21
1758	Receptor binding protein	PhiLj_19
1759	Receptor binding protein	PhiM.16_21
1760	Receptor binding protein	PhiM.5_21
1761	Receptor binding protein	PhiM1127_22
1762	Receptor binding protein	PhiS0139_19
1763	Receptor binding protein	SL4_0020
1764	Receptor binding protein	ViridisJM2_gp19
1765	Receptor binding protein	bIBB29_gp19
1766	Receptor binding protein	bIL170p20
1767	Receptor binding protein	fd13_0020
1768	Receptor binding protein	jj50_ORF18
1769	Receptor binding protein	jm1_0019
1770	Receptor binding protein	p2_0018
1771	Receptor binding protein	phi7_20
1772	Receptor binding protein	sk1p18
1773	Holin	340_0021
1774	Holin	645_0020
1775	Holin	936_0019
1776	Holin	ASCC191_0022
1777	Holin	ASCC273_0022
1778	Holin	ASCC281_0022
1779	Holin	ASCC284_0022
1780	Holin	ASCC287_0022
1781	Holin	ASCC310_0022

1782	Holin	ASCC324_0022
1783	Holin	ASCC337_0022
1784	Holin	ASCC356_0022
1785	Holin	ASCC358_0022
1786	Holin	ASCC365_0022
1787	Holin	ASCC368_0022
1788	Holin	ASCC395_0022
1789	Holin	ASCC397_0022
1790	Holin	ASCC406_0022
1791	Holin	ASCC454_0022
1792	Holin	ASCC460_0022
1793	Holin	ASCC465_0022
1794	Holin	ASCC473_0022
1795	Holin	ASCC476_0022
1796	Holin	ASCC489_0022
1797	Holin	ASCC497_0022
1798	Holin	ASCC502_0022
1799	Holin	ASCC506_0022
1800	Holin	ASCC527_0022
1801	Holin	ASCC531_0022
1802	Holin	ASCC532_0022
1803	Holin	ASCC544_0022
1804	Holin	CB13_0021
1805	Holin	CB14_0021
1806	Holin	CB19_0021
1807	Holin	CB20_0021
1808	Holin	LPPV008_gp21
1809	Holin	LPV712_gp020
1810	Holin	P113G_0020
1811	Holin	P272_0020
1812	Holin	P475_0021
1813	Holin	P680_20
1814	Holin	PastusJM3_20
1815	Holin	Phi10.5_21
1816	Holin	Phi109_23
1817	Holin	Phi114_21
1818	Holin	Phi129_20
1819	Holin	Phi1316_20
1820	Holin	Phi145_22
1821	Holin	Phi155_22
1822	Holin	Phi15_21
1823	Holin	Phi16_23
1824	Holin	Phi17_22
1825	Holin	Phi19.2_20
1826	Holin	Phi19.3_20
1827	Holin	Phi19_20
1828	Holin	Phi4.2_22
1829	Holin	Phi40_23
1830	Holin	Phi43_20
1831	Holin	Phi44_21

1832	Holin	Phi4_21
1833	Holin	Phi5.12_21
1834	Holin	Phi91127_22
1835	Holin	Phi93_24
1836	Holin	PhiA1127_21
1837	Holin	PhiA16_20
1838	Holin	PhiB1127_21
1839	Holin	PhiC0139_21
1840	Holin	PhiD.18_20
1841	Holin	PhiE1127_21
1842	Holin	PhiF.17_21
1843	Holin	PhiF0139_21
1844	Holin	PhiG_21
1845	Holin	PhiJF1_22
1846	Holin	PhiL.18_22
1847	Holin	PhiL.6_22
1848	Holin	PhiLj_20
1849	Holin	PhiM.16_22
1850	Holin	PhiM.5_22
1851	Holin	PhiM1127_23
1852	Holin	PhiS0139_20
1853	Holin	SL4_0021
1854	Holin	ViridisJM2_gp20
1855	Holin	bIBB29_gp20
1856	Holin	bIL170p21
1857	Holin	fd13_0021
1858	Holin	jj50_ORF19
1859	Holin	jm1_0020
1860	Holin	p2_0019
1861	Holin	phi7_21
1862	Holin	sk1p19
1863	Lysin	340_0022
1864	Lysin	645_0021
1865	Lysin	936_0020
1866	Lysin	ASCC191_0023
1867	Lysin	ASCC273_0023
1868	Lysin	ASCC281_0023
1869	Lysin	ASCC284_0023
1870	Lysin	ASCC287_0023
1871	Lysin	ASCC310_0023
1872	Lysin	ASCC324_0023
1873	Lysin	ASCC337_0023
1874	Lysin	ASCC356_0023
1875	Lysin	ASCC358_0023
1876	Lysin	ASCC365_0023
1877	Lysin	ASCC368_0023
1878	Lysin	ASCC395_0023
1879	Lysin	ASCC397_0023
1880	Lysin	ASCC406_0023
1881	Lysin	ASCC454_0023

1882	Lysin	ASCC460_0023
1883	Lysin	ASCC465_0023
1884	Lysin	ASCC473_0023
1885	Lysin	ASCC476_0023
1886	Lysin	ASCC489_0023
1887	Lysin	ASCC497_0023
1888	Lysin	ASCC502_0023
1889	Lysin	ASCC506_0023
1890	Lysin	ASCC527_0023
1891	Lysin	ASCC531_0023
1892	Lysin	ASCC532_0023
1893	Lysin	ASCC544_0023
1894	Lysin	CB13_0022
1895	Lysin	CB14_0022
1896	Lysin	CB19_0022
1897	Lysin	CB20_0022
1898	Lysin	LPPV008_gp22
1899	Lysin	LPV712_gp021
1900	Lysin	P113G_0021
1901	Lysin	P272_0021
1902	Lysin	P475_0022
1903	Lysin	P680_21
1904	Lysin	PastusJM3_21
1905	Lysin	Phi10.5_22
1906	Lysin	Phi109_24
1907	Lysin	Phi114_22
1908	Lysin	Phi129_21
1909	Lysin	Phi1316_21
1910	Lysin	Phi145_23
1911	Lysin	Phi155_23
1912	Lysin	Phi15_22
1913	Lysin	Phi16_24
1914	Lysin	Phi17_23
1915	Lysin	Phi19.2_21
1916	Lysin	Phi19.3_21
1917	Lysin	Phi19_21
1918	Lysin	Phi4.2_23
1919	Lysin	Phi40_24
1920	Lysin	Phi43_21
1921	Lysin	Phi44_22
1922	Lysin	Phi4_22
1923	Lysin	Phi5.12_22
1924	Lysin	Phi91127_23
1925	Lysin	Phi93_25
1926	Lysin	PhiA1127_22
1927	Lysin	PhiA16_21
1928	Lysin	PhiB1127_22
1929	Lysin	PhiC0139_22
1930	Lysin	PhiD.18_21
1931	Lysin	PhiE1127_22

1932	Lysin	PhiF.17_22
1933	Lysin	PhiF0139_22
1934	Lysin	PhiG_22
1935	Lysin	PhiJF1_23
1936	Lysin	PhiL.18_23
1937	Lysin	PhiL.6_23
1938	Lysin	PhiLj_21
1939	Lysin	PhiM.16_23
1940	Lysin	PhiM.5_23
1941	Lysin	PhiM1127_24
1942	Lysin	PhiS0139_21
1943	Lysin	SL4_0022
1944	Lysin	ViridisJM2_gp21
1945	Lysin	bIBB29_gp21
1946	Lysin	bIL170p22
1947	Lysin	fd13_0022
1948	Lysin	jj50_ORF20
1949	Lysin	jm1_0021
1950	Lysin	p2_0020
1951	Lysin	phi7_22
1952	Lysin	sk1p20
1953	Hypothetical protein	340_0026
1954	Hypothetical protein	645_0025
1955	Hypothetical protein	936_0022
1956	Hypothetical protein	ASCC191_0025
1957	Hypothetical protein	ASCC273_0025
1958	Hypothetical protein	ASCC281_0026
1959	Hypothetical protein	ASCC284_0025
1960	Hypothetical protein	ASCC287_0025
1961	Hypothetical protein	ASCC310_0025
1962	Hypothetical protein	ASCC324_0025
1963	Hypothetical protein	ASCC337_0025
1964	Hypothetical protein	ASCC356_0025
1965	Hypothetical protein	ASCC358_0026
1966	Hypothetical protein	ASCC365_0026
1967	Hypothetical protein	ASCC368_0025
1968	Hypothetical protein	ASCC395_0025
1969	Hypothetical protein	ASCC397_0025
1970	Hypothetical protein	ASCC406_0025
1971	Hypothetical protein	ASCC454_0025
1972	Hypothetical protein	ASCC460_0025
1973	Hypothetical protein	ASCC465_0026
1974	Hypothetical protein	ASCC473_0026
1975	Hypothetical protein	ASCC476_0025
1976	Hypothetical protein	ASCC489_0026
1977	Hypothetical protein	ASCC497_0026
1978	Hypothetical protein	ASCC502_0025
1979	Hypothetical protein	ASCC506_0025
1980	Hypothetical protein	ASCC527_0025
1981	Hypothetical protein	ASCC531_0026

1982	Hypothetical protein	ASCC532_0025
1983	Hypothetical protein	ASCC544_0025
1984	Hypothetical protein	CB13_0024
1985	Hypothetical protein	CB14_0024
1986	Hypothetical protein	CB19_0024
1987	Hypothetical protein	CB20_0024
1988	Hypothetical protein	LPPV008_gp25
1989	Hypothetical protein	LPV712_gp024
1990	Hypothetical protein	P113G_0026
1991	Hypothetical protein	P272_0026
1992	Hypothetical protein	P475_0026
1993	Hypothetical protein	P680_25
1994	Hypothetical protein	PastusJM3_24
1995	Hypothetical protein	Phi10.5_26
1996	Hypothetical protein	Phi109_28
1997	Hypothetical protein	Phi114_24
1998	Hypothetical protein	Phi129_23
1999	Hypothetical protein	Phi1316_22
2000	Hypothetical protein	Phi145_27
2001	Hypothetical protein	Phi155_27
2002	Hypothetical protein	Phi15_25
2003	Hypothetical protein	Phi16_28
2004	Hypothetical protein	Phi17_27
2005	Hypothetical protein	Phi19.2_22
2006	Hypothetical protein	Phi19.3_22
2007	Hypothetical protein	Phi19_22
2008	Hypothetical protein	Phi4.2_27
2009	Hypothetical protein	Phi40_28
2010	Hypothetical protein	Phi43_23
2011	Hypothetical protein	Phi44_25
2012	Hypothetical protein	Phi4_23
2013	Hypothetical protein	Phi5.12_24
2014	Hypothetical protein	Phi91127_27
2015	Hypothetical protein	Phi93_29
2016	Hypothetical protein	PhiA1127_23
2017	Hypothetical protein	PhiA16_22
2018	Hypothetical protein	PhiB1127_23
2019	Hypothetical protein	PhiC0139_23
2020	Hypothetical protein	PhiD.18_23
2021	Hypothetical protein	PhiE1127_25
2022	Hypothetical protein	PhiF.17_24
2023	Hypothetical protein	PhiF0139_24
2024	Hypothetical protein	PhiG_24
2025	Hypothetical protein	PhiJF1_25
2026	Hypothetical protein	PhiL.18_27
2027	Hypothetical protein	PhiL.6_27
2028	Hypothetical protein	PhiLj_22
2029	Hypothetical protein	PhiM.16_26
2030	Hypothetical protein	PhiM.5_27
2031	Hypothetical protein	PhiM1127_28

2032	Hypothetical protein	PhiS0139_22
2033	Hypothetical protein	SL4_0025
2034	Hypothetical protein	ViridisJM2_gp23
2035	Hypothetical protein	bIBB29_gp25
2036	Hypothetical protein	bIL170p27
2037	Hypothetical protein	fd13_0024
2038	Hypothetical protein	jj50_ORF21
2039	Hypothetical protein	jm1_0023
2040	Hypothetical protein	p2_0022
2041	Hypothetical protein	phi7_25
2042	Hypothetical protein	sk1p22
2043	Hypothetical protein	340_0027
2044	Hypothetical protein	645_0026
2045	Hypothetical protein	936_0024
2046	Hypothetical protein	ASCC191_0026
2047	Hypothetical protein	ASCC273_0026
2048	Hypothetical protein	ASCC281_0028
2049	Hypothetical protein	ASCC284_0027
2050	Hypothetical protein	ASCC287_0026
2051	Hypothetical protein	ASCC310_0027
2052	Hypothetical protein	ASCC324_0026
2053	Hypothetical protein	ASCC337_0026
2054	Hypothetical protein	ASCC356_0027
2055	Hypothetical protein	ASCC358_0028
2056	Hypothetical protein	ASCC365_0028
2057	Hypothetical protein	ASCC368_0026
2058	Hypothetical protein	ASCC395_0026
2059	Hypothetical protein	ASCC397_0026
2060	Hypothetical protein	ASCC406_0026
2061	Hypothetical protein	ASCC454_0026
2062	Hypothetical protein	ASCC460_0026
2063	Hypothetical protein	ASCC465_0028
2064	Hypothetical protein	ASCC473_0028
2065	Hypothetical protein	ASCC476_0026
2066	Hypothetical protein	ASCC489_0027
2067	Hypothetical protein	ASCC497_0028
2068	Hypothetical protein	ASCC502_0026
2069	Hypothetical protein	ASCC506_0026
2070	Hypothetical protein	ASCC527_0026
2071	Hypothetical protein	ASCC531_0028
2072	Hypothetical protein	ASCC532_0027
2073	Hypothetical protein	ASCC544_0026
2074	Hypothetical protein	CB13_0025
2075	Hypothetical protein	CB14_0025
2076	Hypothetical protein	CB19_0025
2077	Hypothetical protein	CB20_0025
2078	Hypothetical protein	LPPV008_gp26
2079	Hypothetical protein	LPV712_gp026
2080	Hypothetical protein	P113G_0027
2081	Hypothetical protein	P272_0027

2082	Hypothetical protein	P475_0027
2083	Hypothetical protein	P680_26
2084	Hypothetical protein	PastusJM3_25
2085	Hypothetical protein	Phi10.5_28
2086	Hypothetical protein	Phi109_31
2087	Hypothetical protein	Phi114_26
2088	Hypothetical protein	Phi129_25
2089	Hypothetical protein	Phi1316_24
2090	Hypothetical protein	Phi145_30
2091	Hypothetical protein	Phi155_29
2092	Hypothetical protein	Phi15_27
2093	Hypothetical protein	Phi16_30
2094	Hypothetical protein	Phi17_29
2095	Hypothetical protein	Phi19.2_24
2096	Hypothetical protein	Phi19.3_24
2097	Hypothetical protein	Phi19_24
2098	Hypothetical protein	Phi4.2_29
2099	Hypothetical protein	Phi40_30
2100	Hypothetical protein	Phi43_25
2101	Hypothetical protein	Phi44_26
2102	Hypothetical protein	Phi4_25
2103	Hypothetical protein	Phi5.12_25
2104	Hypothetical protein	Phi91127_29
2105	Hypothetical protein	Phi93_32
2106	Hypothetical protein	PhiA1127_25
2107	Hypothetical protein	PhiA16_24
2108	Hypothetical protein	PhiB1127_25
2109	Hypothetical protein	PhiC0139_25
2110	Hypothetical protein	PhiD.18_25
2111	Hypothetical protein	PhiE1127_27
2112	Hypothetical protein	PhiF.17_26
2113	Hypothetical protein	PhiF0139_26
2114	Hypothetical protein	PhiG_26
2115	Hypothetical protein	PhiJF1_27
2116	Hypothetical protein	PhiL.18_29
2117	Hypothetical protein	PhiL.6_29
2118	Hypothetical protein	PhiLj_24
2119	Hypothetical protein	PhiM.16_29
2120	Hypothetical protein	PhiM.5_29
2121	Hypothetical protein	PhiM1127_30
2122	Hypothetical protein	PhiS0139_24
2123	Hypothetical protein	SL4_0026
2124	Hypothetical protein	ViridisJM2_gp24
2125	Hypothetical protein	bIBB29_gp26b
2126	Hypothetical protein	bIBB29_gp27
2127	Hypothetical protein	bIL170p29
2128	Hypothetical protein	fd13_0026
2129	Hypothetical protein	jj50_ORF22
2130	Hypothetical protein	jm1_0025
2131	Hypothetical protein	p2_0023

2132	Hypothetical protein	phi7_26
2133	Hypothetical protein	sk1p24
2134	Hypothetical protein	340_0040
2135	Hypothetical protein	645_0036
2136	Hypothetical protein	936_0035
2137	Hypothetical protein	ASCC191_0041
2138	Hypothetical protein	ASCC273_0040
2139	Hypothetical protein	ASCC281_0040
2140	Hypothetical protein	ASCC284_0040
2141	Hypothetical protein	ASCC287_0040
2142	Hypothetical protein	ASCC310_0040
2143	Hypothetical protein	ASCC324_0040
2144	Hypothetical protein	ASCC337_0040
2145	Hypothetical protein	ASCC356_0040
2146	Hypothetical protein	ASCC358_0040
2147	Hypothetical protein	ASCC365_0040
2148	Hypothetical protein	ASCC368_0040
2149	Hypothetical protein	ASCC395_0040
2150	Hypothetical protein	ASCC397_0040
2151	Hypothetical protein	ASCC406_0040
2152	Hypothetical protein	ASCC454_0040
2153	Hypothetical protein	ASCC460_0040
2154	Hypothetical protein	ASCC465_0041
2155	Hypothetical protein	ASCC473_0041
2156	Hypothetical protein	ASCC476_0040
2157	Hypothetical protein	ASCC489_0040
2158	Hypothetical protein	ASCC497_0041
2159	Hypothetical protein	ASCC502_0040
2160	Hypothetical protein	ASCC506_0040
2161	Hypothetical protein	ASCC527_0040
2162	Hypothetical protein	ASCC531_0041
2163	Hypothetical protein	ASCC532_0040a
2164	Hypothetical protein	ASCC544_0040
2165	Hypothetical protein	CB13_0039
2166	Hypothetical protein	CB14_0036
2167	Hypothetical protein	CB19_0036
2168	Hypothetical protein	CB20_0036
2169	Hypothetical protein	LPPV008_gp37
2170	Hypothetical protein	LPV712_gp032
2171	Hypothetical protein	P113G_0038
2172	Hypothetical protein	P272_0040
2173	Hypothetical protein	P475_0039
2174	Hypothetical protein	P680_36
2175	Hypothetical protein	PastusJM3_34
2176	Hypothetical protein	Phi10.5_45
2177	Hypothetical protein	Phi109_40
2178	Hypothetical protein	Phi114_39
2179	Hypothetical protein	Phi129_36
2180	Hypothetical protein	Phi1316_38
2181	Hypothetical protein	Phi145_39

2182	Hypothetical protein	Phi155_38
2183	Hypothetical protein	Phi15_40
2184	Hypothetical protein	Phi16_40
2185	Hypothetical protein	Phi17_39
2186	Hypothetical protein	Phi19.2_39
2187	Hypothetical protein	Phi19.3_37
2188	Hypothetical protein	Phi19_33
2189	Hypothetical protein	Phi4.2_41
2190	Hypothetical protein	Phi40_40
2191	Hypothetical protein	Phi43_36
2192	Hypothetical protein	Phi44_39
2193	Hypothetical protein	Phi4_35
2194	Hypothetical protein	Phi5.12_37
2195	Hypothetical protein	Phi91127_40
2196	Hypothetical protein	Phi93_41
2197	Hypothetical protein	PhiA1127_36
2198	Hypothetical protein	PhiA16_35
2199	Hypothetical protein	PhiB1127_38
2200	Hypothetical protein	PhiC0139_38
2201	Hypothetical protein	PhiD.18_36
2202	Hypothetical protein	PhiE1127_40
2203	Hypothetical protein	PhiF.17_40
2204	Hypothetical protein	PhiF0139_40
2205	Hypothetical protein	PhiG_39
2206	Hypothetical protein	PhiG_40
2207	Hypothetical protein	PhiJF1_40
2208	Hypothetical protein	PhiL.18_39
2209	Hypothetical protein	PhiL.6_42
2210	Hypothetical protein	PhiLj_34
2211	Hypothetical protein	PhiM.16_39
2212	Hypothetical protein	PhiM.5_41
2213	Hypothetical protein	PhiM1127_43
2214	Hypothetical protein	PhiS0139_36
2215	Hypothetical protein	SL4_0036
2216	Hypothetical protein	ViridisJM2_gp39
2217	Hypothetical protein	bIBB29_gp39
2218	Hypothetical protein	bIL170p45
2219	Hypothetical protein	fd13_0036
2220	Hypothetical protein	jj50_ORF31
2221	Hypothetical protein	jm1_0036
2222	Hypothetical protein	p2_0032
2223	Hypothetical protein	phi7_38
2224	Hypothetical protein	sk1p34
2225	Hypothetical protein	340_0041
2226	Hypothetical protein	645_0037
2227	Hypothetical protein	936_0036
2228	Hypothetical protein	ASCC191_0043
2229	Hypothetical protein	ASCC273_0042
2230	Hypothetical protein	ASCC281_0041
2231	Hypothetical protein	ASCC284_0041

2232	Hypothetical protein	ASCC287_0042
2233	Hypothetical protein	ASCC310_0041
2234	Hypothetical protein	ASCC324_0042
2235	Hypothetical protein	ASCC337_0042
2236	Hypothetical protein	ASCC356_0041
2237	Hypothetical protein	ASCC358_0041
2238	Hypothetical protein	ASCC365_0041
2239	Hypothetical protein	ASCC368_0042
2240	Hypothetical protein	ASCC395_0042
2241	Hypothetical protein	ASCC397_0042
2242	Hypothetical protein	ASCC406_0042
2243	Hypothetical protein	ASCC454_0042
2244	Hypothetical protein	ASCC460_0042
2245	Hypothetical protein	ASCC465_0042
2246	Hypothetical protein	ASCC473_0042
2247	Hypothetical protein	ASCC476_0042
2248	Hypothetical protein	ASCC489_0041
2249	Hypothetical protein	ASCC497_0042
2250	Hypothetical protein	ASCC502_0042
2251	Hypothetical protein	ASCC506_0042
2252	Hypothetical protein	ASCC527_0042
2253	Hypothetical protein	ASCC531_0042
2254	Hypothetical protein	ASCC532_0043
2255	Hypothetical protein	ASCC544_0042
2256	Hypothetical protein	CB13_0040
2257	Hypothetical protein	CB14_0037
2258	Hypothetical protein	CB19_0037
2259	Hypothetical protein	CB20_0037
2260	Hypothetical protein	LPPV008_gp38
2261	Hypothetical protein	LPV712_gp033
2262	Hypothetical protein	P113G_0039
2263	Hypothetical protein	P272_0041
2264	Hypothetical protein	P475_0040
2265	Hypothetical protein	P680_37
2266	Hypothetical protein	PastusJM3_35
2267	Hypothetical protein	Phi10.5_46
2268	Hypothetical protein	Phi109_41
2269	Hypothetical protein	Phi114_40
2270	Hypothetical protein	Phi129_37
2271	Hypothetical protein	Phi1316_39
2272	Hypothetical protein	Phi145_40
2273	Hypothetical protein	Phi155_39
2274	Hypothetical protein	Phi15_41
2275	Hypothetical protein	Phi16_41
2276	Hypothetical protein	Phi17_40
2277	Hypothetical protein	Phi19.2_40
2278	Hypothetical protein	Phi19.3_38
2279	Hypothetical protein	Phi19_34
2280	Hypothetical protein	Phi4.2_42
2281	Hypothetical protein	Phi40_41

2282	Hypothetical protein	Phi43_37
2283	Hypothetical protein	Phi44_40
2284	Hypothetical protein	Phi4_36
2285	Hypothetical protein	Phi5.12_38
2286	Hypothetical protein	Phi91127_41
2287	Hypothetical protein	Phi93_42
2288	Hypothetical protein	PhiA1127_37
2289	Hypothetical protein	PhiA16_36
2290	Hypothetical protein	PhiB1127_39
2291	Hypothetical protein	PhiC0139_39
2292	Hypothetical protein	PhiD.18_37
2293	Hypothetical protein	PhiE1127_41
2294	Hypothetical protein	PhiF.17_41
2295	Hypothetical protein	PhiF0139_41
2296	Hypothetical protein	PhiG_41
2297	Hypothetical protein	PhiJF1_41
2298	Hypothetical protein	PhiL.18_40
2299	Hypothetical protein	PhiL.6_43
2300	Hypothetical protein	PhiLj_35
2301	Hypothetical protein	PhiM.16_40
2302	Hypothetical protein	PhiM.5_42
2303	Hypothetical protein	PhiM1127_44
2304	Hypothetical protein	PhiS0139_37
2305	Hypothetical protein	SL4_0037
2306	Hypothetical protein	ViridisJM2_gp40
2307	Hypothetical protein	bIBB29_gp40
2308	Hypothetical protein	bIL170p46
2309	Hypothetical protein	fd13_0037
2310	Hypothetical protein	jj50_ORF32
2311	Hypothetical protein	jm1_0037
2312	Hypothetical protein	p2_0033
2313	Hypothetical protein	phi7_39
2314	Hypothetical protein	sk1p35
2315	Single stranded DNA binding protein	340_0042
2316	Single stranded DNA binding protein	645_0038
2317	Single stranded DNA binding protein	936_0037
2318	Single stranded DNA binding protein	ASCC191_0044
2319	Single stranded DNA binding protein	ASCC273_0043
2320	Single stranded DNA binding protein	ASCC281_0042
2321	Single stranded DNA binding protein	ASCC284_0042
2322	Single stranded DNA binding protein	ASCC287_0043
2323	Single stranded DNA binding protein	ASCC310_0042
2324	Single stranded DNA binding protein	ASCC324_0043
2325	Single stranded DNA binding protein	ASCC337_0043
2326	Single stranded DNA binding protein	ASCC356_0042
2327	Single stranded DNA binding protein	ASCC358_0042
2328	Single stranded DNA binding protein	ASCC365_0042
2329	Single stranded DNA binding protein	ASCC368_0043
2330	Single stranded DNA binding protein	ASCC395_0043
2331	Single stranded DNA binding protein	ASCC397_0043

2332 Single stranded DNA binding proteinASCC406_0043
2333 Single stranded DNA binding proteinASCC454_0043
2334 Single stranded DNA binding proteinASCC460_0043
2335 Single stranded DNA binding proteinASCC465_0043
2336 Single stranded DNA binding proteinASCC473_0043
2337 Single stranded DNA binding proteinASCC476_0043
2338 Single stranded DNA binding proteinASCC489_0042
2339 Single stranded DNA binding proteinASCC497_0043
2340 Single stranded DNA binding proteinASCC502_0043
2341 Single stranded DNA binding proteinASCC506_0043
2342 Single stranded DNA binding proteinASCC527_0043
2343 Single stranded DNA binding proteinASCC531_0043
2344 Single stranded DNA binding proteinASCC532_0044
2345 Single stranded DNA binding proteinASCC544_0043
2346 Single stranded DNA binding proteinCB13_0041
2347 Single stranded DNA binding proteinCB14_0038
2348 Single stranded DNA binding proteinCB19_0038
2349 Single stranded DNA binding proteinCB20_0038
2350 Single stranded DNA binding proteinLPPV008_gp39
2351 Single stranded DNA binding proteinLPV712_gp034
2352 Single stranded DNA binding proteinP113G_0040
2353 Single stranded DNA binding proteinP272_0042
2354 Single stranded DNA binding proteinP475_0041
2355 Single stranded DNA binding proteinP680_38
2356 Single stranded DNA binding proteinPastusJM3_36
2357 Single stranded DNA binding proteinPhi10.5_47
2358 Single stranded DNA binding proteinPhi109_42
2359 Single stranded DNA binding proteinPhi114_41
2360 Single stranded DNA binding proteinPhi129_38
2361 Single stranded DNA binding proteinPhi1316_40
2362 Single stranded DNA binding proteinPhi145_41
2363 Single stranded DNA binding proteinPhi155_40
2364 Single stranded DNA binding proteinPhi15_42
2365 Single stranded DNA binding proteinPhi16_42
2366 Single stranded DNA binding proteinPhi17_41
2367 Single stranded DNA binding proteinPhi19.2_41
2368 Single stranded DNA binding proteinPhi19.3_39
2369 Single stranded DNA binding proteinPhi19_35
2370 Single stranded DNA binding proteinPhi4.2_43
2371 Single stranded DNA binding proteinPhi40_42
2372 Single stranded DNA binding proteinPhi43_38
2373 Single stranded DNA binding proteinPhi44_41
2374 Single stranded DNA binding proteinPhi4_37
2375 Single stranded DNA binding proteinPhi5.12_39
2376 Single stranded DNA binding proteinPhi91127_42
2377 Single stranded DNA binding proteinPhi93_43
2378 Single stranded DNA binding proteinPhiA1127_38
2379 Single stranded DNA binding proteinPhiA16_37
2380 Single stranded DNA binding proteinPhiB1127_40
2381 Single stranded DNA binding proteinPhiC0139_40

2382 Single stranded DNA binding proteinPhiD.18_38
2383 Single stranded DNA binding proteinPhiE1127_42
2384 Single stranded DNA binding proteinPhiF.17_42
2385 Single stranded DNA binding proteinPhiF0139_42
2386 Single stranded DNA binding proteinPhiG_42
2387 Single stranded DNA binding proteinPhiJF1_42
2388 Single stranded DNA binding proteinPhiL.18_41
2389 Single stranded DNA binding proteinPhiL.6_44
2390 Single stranded DNA binding proteinPhiLj_36
2391 Single stranded DNA binding proteinPhiM.16_41
2392 Single stranded DNA binding proteinPhiM.5_43
2393 Single stranded DNA binding proteinPhiM1127_45
2394 Single stranded DNA binding proteinPhiS0139_38
2395 Single stranded DNA binding proteinSL4_0038
2396 Single stranded DNA binding proteinViridisJM2_gp41
2397 Single stranded DNA binding proteinbIBB29_gp41
2398 Single stranded DNA binding proteinbIL170p47
2399 Single stranded DNA binding proteinfd13_0038
2400 Single stranded DNA binding proteinjj50_ORF33
2401 Single stranded DNA binding proteinjm1_0038
2402 Single stranded DNA binding proteinp2_0034
2403 Single stranded DNA binding proteinphi7_40
2404 Single stranded DNA binding proteinsk1p36
2405 Homologous reombinase Sak 340_0043
2406 Homologous reombinase Sak 645_0039
2407 Homologous reombinase Sak 936_0038
2408 Homologous reombinase Sak ASCC191_0045
2409 Homologous reombinase Sak ASCC273_0044
2410 Homologous reombinase Sak ASCC281_0043
2411 Homologous reombinase Sak ASCC284_0043
2412 Homologous reombinase Sak ASCC287_0044
2413 Homologous reombinase Sak ASCC310_0043
2414 Homologous reombinase Sak ASCC324_0044
2415 Homologous reombinase Sak ASCC337_0044
2416 Homologous reombinase Sak ASCC356_0043
2417 Homologous reombinase Sak ASCC358_0043
2418 Homologous reombinase Sak ASCC365_0043
2419 Homologous reombinase Sak ASCC368_0044
2420 Homologous reombinase Sak ASCC395_0044
2421 Homologous reombinase Sak ASCC397_0044
2422 Homologous reombinase Sak ASCC406_0044
2423 Homologous reombinase Sak ASCC454_0044
2424 Homologous reombinase Sak ASCC460_0044
2425 Homologous reombinase Sak ASCC465_0044
2426 Homologous reombinase Sak ASCC473_0044
2427 Homologous reombinase Sak ASCC476_0044
2428 Homologous reombinase Sak ASCC489_0043
2429 Homologous reombinase Sak ASCC497_0044
2430 Homologous reombinase Sak ASCC502_0044
2431 Homologous reombinase Sak ASCC506_0044

2432	Homologous reombinase Sak ASCC527_0044
2433	Homologous reombinase Sak ASCC531_0044
2434	Homologous reombinase Sak ASCC532_0045
2435	Homologous reombinase Sak ASCC544_0044
2436	Homologous reombinase Sak CB13_0042
2437	Homologous reombinase Sak CB14_0039
2438	Homologous reombinase Sak CB19_0039
2439	Homologous reombinase Sak CB20_0039
2440	Homologous reombinase Sak LPPV008_gp40
2441	Homologous reombinase Sak LPV712_gp035
2442	Homologous reombinase Sak P113G_0041
2443	Homologous reombinase Sak P272_0043
2444	Homologous reombinase Sak P475_0042
2445	Homologous reombinase Sak P680_39
2446	Homologous reombinase Sak PastusJM3_37
2447	Homologous reombinase Sak Phi10.5_48
2448	Homologous reombinase Sak Phi109_43
2449	Homologous reombinase Sak Phi114_42
2450	Homologous reombinase Sak Phi129_39
2451	Homologous reombinase Sak Phi1316_41
2452	Homologous reombinase Sak Phi145_42
2453	Homologous reombinase Sak Phi155_41
2454	Homologous reombinase Sak Phi15_43
2455	Homologous reombinase Sak Phi16_43
2456	Homologous reombinase Sak Phi17_42
2457	Homologous reombinase Sak Phi19.2_42
2458	Homologous reombinase Sak Phi19.3_40
2459	Homologous reombinase Sak Phi19_36
2460	Homologous reombinase Sak Phi4.2_44
2461	Homologous reombinase Sak Phi40_43
2462	Homologous reombinase Sak Phi43_39
2463	Homologous reombinase Sak Phi44_42
2464	Homologous reombinase Sak Phi4_38
2465	Homologous reombinase Sak Phi5.12_40
2466	Homologous reombinase Sak Phi91127_43
2467	Homologous reombinase Sak Phi93_44
2468	Homologous reombinase Sak PhiA1127_39
2469	Homologous reombinase Sak PhiA16_38
2470	Homologous reombinase Sak PhiB1127_41
2471	Homologous reombinase Sak PhiC0139_41
2472	Homologous reombinase Sak PhiD.18_39
2473	Homologous reombinase Sak PhiE1127_43
2474	Homologous reombinase Sak PhiF.17_43
2475	Homologous reombinase Sak PhiF0139_43
2476	Homologous reombinase Sak PhiG_43
2477	Homologous reombinase Sak PhiJF1_43
2478	Homologous reombinase Sak PhiL.18_42
2479	Homologous reombinase Sak PhiL.6_45
2480	Homologous reombinase Sak PhiLj_37
2481	Homologous reombinase Sak PhiM.16_42

2482	Homologous reombinase Sak	PhiM.5_44
2483	Homologous reombinase Sak	PhiM1127_46
2484	Homologous reombinase Sak	PhiS0139_39
2485	Homologous reombinase Sak	SL4_0039
2486	Homologous reombinase Sak	ViridisJM2_gp42
2487	Homologous reombinase Sak	bIBB29_gp42
2488	Homologous reombinase Sak	bIL170p48
2489	Homologous reombinase Sak	fd13_0039
2490	Homologous reombinase Sak	jj50_ORF34
2491	Homologous reombinase Sak	jm1_0039
2492	Homologous reombinase Sak	p2_0035
2493	Homologous reombinase Sak	phi7_41
2494	Homologous reombinase Sak	sk1p37
2495	Hypothetical protein	340_0045
2496	Hypothetical protein	645_0041
2497	Hypothetical protein	936_0039
2498	Hypothetical protein	ASCC191_0047
2499	Hypothetical protein	ASCC273_0046
2500	Hypothetical protein	ASCC281_0045
2501	Hypothetical protein	ASCC284_0045
2502	Hypothetical protein	ASCC287_0046
2503	Hypothetical protein	ASCC310_0045
2504	Hypothetical protein	ASCC324_0046
2505	Hypothetical protein	ASCC337_0046
2506	Hypothetical protein	ASCC356_0045
2507	Hypothetical protein	ASCC358_0045
2508	Hypothetical protein	ASCC365_0045
2509	Hypothetical protein	ASCC368_0046
2510	Hypothetical protein	ASCC395_0046
2511	Hypothetical protein	ASCC397_0046
2512	Hypothetical protein	ASCC406_0046
2513	Hypothetical protein	ASCC454_0046
2514	Hypothetical protein	ASCC460_0046
2515	Hypothetical protein	ASCC465_0046
2516	Hypothetical protein	ASCC473_0046
2517	Hypothetical protein	ASCC476_0046
2518	Hypothetical protein	ASCC489_0044
2519	Hypothetical protein	ASCC497_0046
2520	Hypothetical protein	ASCC502_0046
2521	Hypothetical protein	ASCC506_0046
2522	Hypothetical protein	ASCC527_0046
2523	Hypothetical protein	ASCC531_0047
2524	Hypothetical protein	ASCC532_0047
2525	Hypothetical protein	ASCC544_0046
2526	Hypothetical protein	CB13_0043
2527	Hypothetical protein	CB14_0041
2528	Hypothetical protein	CB19_0041
2529	Hypothetical protein	CB20_0041
2530	Hypothetical protein	LPPV008_gp42
2531	Hypothetical protein	LPV712_gp038

2532	Hypothetical protein	P113G_0043
2533	Hypothetical protein	P272_0045
2534	Hypothetical protein	P475_0043a
2535	Hypothetical protein	P680_40
2536	Hypothetical protein	PastusJM3_39
2537	Hypothetical protein	Phi10.5_50
2538	Hypothetical protein	Phi109_44
2539	Hypothetical protein	Phi114_43
2540	Hypothetical protein	Phi129_40
2541	Hypothetical protein	Phi1316_42
2542	Hypothetical protein	Phi145_44
2543	Hypothetical protein	Phi155_43
2544	Hypothetical protein	Phi15_46
2545	Hypothetical protein	Phi16_45
2546	Hypothetical protein	Phi17_44
2547	Hypothetical protein	Phi19.2_43
2548	Hypothetical protein	Phi19.3_41
2549	Hypothetical protein	Phi19_38
2550	Hypothetical protein	Phi4.2_46
2551	Hypothetical protein	Phi40_45
2552	Hypothetical protein	Phi43_41
2553	Hypothetical protein	Phi44_43
2554	Hypothetical protein	Phi4_40
2555	Hypothetical protein	Phi5.12_42
2556	Hypothetical protein	Phi91127_45
2557	Hypothetical protein	Phi93_45
2558	Hypothetical protein	PhiA1127_41
2559	Hypothetical protein	PhiA16_39
2560	Hypothetical protein	PhiB1127_42
2561	Hypothetical protein	PhiC0139_42
2562	Hypothetical protein	PhiD.18_40
2563	Hypothetical protein	PhiE1127_45
2564	Hypothetical protein	PhiF.17_45
2565	Hypothetical protein	PhiF0139_45
2566	Hypothetical protein	PhiG_45
2567	Hypothetical protein	PhiJF1_46
2568	Hypothetical protein	PhiL.18_44
2569	Hypothetical protein	PhiL.6_47
2570	Hypothetical protein	PhiLj_38
2571	Hypothetical protein	PhiM.16_43
2572	Hypothetical protein	PhiM.5_46
2573	Hypothetical protein	PhiM1127_47
2574	Hypothetical protein	PhiS0139_41
2575	Hypothetical protein	SL4_0040
2576	Hypothetical protein	ViridisJM2_gp43
2577	Hypothetical protein	bIBB29_gp43
2578	Hypothetical protein	bIL170p50
2579	Hypothetical protein	fd13_0041
2580	Hypothetical protein	jj50_ORF37
2581	Hypothetical protein	jm1_0041

2582	Hypothetical protein	p2_0038
2583	Hypothetical protein	phi7_43
2584	Hypothetical protein	sk1p40
2585	Middle expressed protein 2	340_0055
2586	Middle expressed protein 2	645_0047
2587	Middle expressed protein 2	936_0046
2588	Middle expressed protein 2	ASCC191_0058
2589	Middle expressed protein 2	ASCC273_0057
2590	Middle expressed protein 2	ASCC281_0055
2591	Middle expressed protein 2	ASCC284_0056
2592	Middle expressed protein 2	ASCC287_0057
2593	Middle expressed protein 2	ASCC310_0054
2594	Middle expressed protein 2	ASCC324_0057
2595	Middle expressed protein 2	ASCC337_0057
2596	Middle expressed protein 2	ASCC356_0053
2597	Middle expressed protein 2	ASCC358_0055
2598	Middle expressed protein 2	ASCC365_0055
2599	Middle expressed protein 2	ASCC368_0057
2600	Middle expressed protein 2	ASCC395_0057
2601	Middle expressed protein 2	ASCC397_0057
2602	Middle expressed protein 2	ASCC406_0057
2603	Middle expressed protein 2	ASCC454_0057
2604	Middle expressed protein 2	ASCC460_0057
2605	Middle expressed protein 2	ASCC465_0055
2606	Middle expressed protein 2	ASCC473_0055
2607	Middle expressed protein 2	ASCC476_0057
2608	Middle expressed protein 2	ASCC489_0053
2609	Middle expressed protein 2	ASCC497_0055
2610	Middle expressed protein 2	ASCC502_0057
2611	Middle expressed protein 2	ASCC506_0057
2612	Middle expressed protein 2	ASCC527_0057
2613	Middle expressed protein 2	ASCC531_0056
2614	Middle expressed protein 2	ASCC532_0056
2615	Middle expressed protein 2	ASCC544_0057
2616	Middle expressed protein 2	CB13_0052
2617	Middle expressed protein 2	CB14_0049
2618	Middle expressed protein 2	CB19_0048
2619	Middle expressed protein 2	CB20_0048
2620	Middle expressed protein 2	LPPV008_gp54
2621	Middle expressed protein 2	LPV712_gp052
2622	Middle expressed protein 2	P113G_0054
2623	Middle expressed protein 2	P272_0057
2624	Middle expressed protein 2	P475_0054
2625	Middle expressed protein 2	P680_48
2626	Middle expressed protein 2	PastusJM3_47
2627	Middle expressed protein 2	Phi10.5_61
2628	Middle expressed protein 2	Phi109_52
2629	Middle expressed protein 2	Phi114_51
2630	Middle expressed protein 2	Phi129_52
2631	Middle expressed protein 2	Phi1316_52

2632	Middle expressed protein 2	Phi145_52
2633	Middle expressed protein 2	Phi155_52
2634	Middle expressed protein 2	Phi15_53
2635	Middle expressed protein 2	Phi16_54
2636	Middle expressed protein 2	Phi17_54
2637	Middle expressed protein 2	Phi19.2_53
2638	Middle expressed protein 2	Phi19.3_50
2639	Middle expressed protein 2	Phi19_48
2640	Middle expressed protein 2	Phi4.2_56
2641	Middle expressed protein 2	Phi40_54
2642	Middle expressed protein 2	Phi43_52
2643	Middle expressed protein 2	Phi44_50
2644	Middle expressed protein 2	Phi4_49
2645	Middle expressed protein 2	Phi5.12_51
2646	Middle expressed protein 2	Phi91127_54
2647	Middle expressed protein 2	Phi93_53
2648	Middle expressed protein 2	PhiA1127_50
2649	Middle expressed protein 2	PhiA16_47
2650	Middle expressed protein 2	PhiB1127_51
2651	Middle expressed protein 2	PhiC0139_51
2652	Middle expressed protein 2	PhiD.18_51
2653	Middle expressed protein 2	PhiE1127_57
2654	Middle expressed protein 2	PhiF.17_54
2655	Middle expressed protein 2	PhiF0139_54
2656	Middle expressed protein 2	PhiG_55
2657	Middle expressed protein 2	PhiJF1_55
2658	Middle expressed protein 2	PhiL.18_53
2659	Middle expressed protein 2	PhiL.6_56
2660	Middle expressed protein 2	PhiLj_47
2661	Middle expressed protein 2	PhiM.16_52
2662	Middle expressed protein 2	PhiM.5_55
2663	Middle expressed protein 2	PhiM1127_57
2664	Middle expressed protein 2	PhiS0139_51
2665	Middle expressed protein 2	SL4_0049
2666	Middle expressed protein 2	ViridisJM2_gp57
2667	Middle expressed protein 2	bIBB29_gp52
2668	Middle expressed protein 2	bIL170p62
2669	Middle expressed protein 2	fd13_0051
2670	Middle expressed protein 2	jj50_ORF47
2671	Middle expressed protein 2	jm1_0049
2672	Middle expressed protein 2	p2_0047
2673	Middle expressed protein 2	phi7_54
2674	Middle expressed protein 2	sk1p54
2675	RuvC-Like holliday endonuclease	340_0056
2676	RuvC-Like holliday endonuclease	645_0048
2677	RuvC-Like holliday endonuclease	936_0047
2678	RuvC-Like holliday endonuclease	ASCC191_0059
2679	RuvC-Like holliday endonuclease	ASCC273_0058
2680	RuvC-Like holliday endonuclease	ASCC281_0056
2681	RuvC-Like holliday endonuclease	ASCC284_0057

2682	RuvC-Like holliday endonuclease	ASCC287_0058
2683	RuvC-Like holliday endonuclease	ASCC310_0055
2684	RuvC-Like holliday endonuclease	ASCC324_0058
2685	RuvC-Like holliday endonuclease	ASCC337_0058
2686	RuvC-Like holliday endonuclease	ASCC356_0054
2687	RuvC-Like holliday endonuclease	ASCC358_0056
2688	RuvC-Like holliday endonuclease	ASCC365_0056
2689	RuvC-Like holliday endonuclease	ASCC368_0058
2690	RuvC-Like holliday endonuclease	ASCC395_0058
2691	RuvC-Like holliday endonuclease	ASCC397_0058
2692	RuvC-Like holliday endonuclease	ASCC406_0058
2693	RuvC-Like holliday endonuclease	ASCC454_0058
2694	RuvC-Like holliday endonuclease	ASCC460_0058
2695	RuvC-Like holliday endonuclease	ASCC465_0056
2696	RuvC-Like holliday endonuclease	ASCC473_0056
2697	RuvC-Like holliday endonuclease	ASCC476_0058
2698	RuvC-Like holliday endonuclease	ASCC489_0054
2699	RuvC-Like holliday endonuclease	ASCC497_0056
2700	RuvC-Like holliday endonuclease	ASCC502_0058
2701	RuvC-Like holliday endonuclease	ASCC506_0058
2702	RuvC-Like holliday endonuclease	ASCC527_0058
2703	RuvC-Like holliday endonuclease	ASCC531_0057
2704	RuvC-Like holliday endonuclease	ASCC532_0057
2705	RuvC-Like holliday endonuclease	ASCC544_0058
2706	RuvC-Like holliday endonuclease	CB13_0053
2707	RuvC-Like holliday endonuclease	CB14_0050
2708	RuvC-Like holliday endonuclease	CB19_0049
2709	RuvC-Like holliday endonuclease	CB20_0049
2710	RuvC-Like holliday endonuclease	LPPV008_gp55
2711	RuvC-Like holliday endonuclease	LPV712_gp053
2712	RuvC-Like holliday endonuclease	P113G_0055
2713	RuvC-Like holliday endonuclease	P272_0058
2714	RuvC-Like holliday endonuclease	P475_0055
2715	RuvC-Like holliday endonuclease	P680_49
2716	RuvC-Like holliday endonuclease	PastusJM3_48
2717	RuvC-Like holliday endonuclease	Phi10.5_62
2718	RuvC-Like holliday endonuclease	Phi109_53
2719	RuvC-Like holliday endonuclease	Phi114_52
2720	RuvC-Like holliday endonuclease	Phi129_53
2721	RuvC-Like holliday endonuclease	Phi1316_53
2722	RuvC-Like holliday endonuclease	Phi145_53
2723	RuvC-Like holliday endonuclease	Phi155_53
2724	RuvC-Like holliday endonuclease	Phi15_54
2725	RuvC-Like holliday endonuclease	Phi16_55
2726	RuvC-Like holliday endonuclease	Phi17_55
2727	RuvC-Like holliday endonuclease	Phi19.2_54
2728	RuvC-Like holliday endonuclease	Phi19.3_51
2729	RuvC-Like holliday endonuclease	Phi19_49
2730	RuvC-Like holliday endonuclease	Phi4.2_57
2731	RuvC-Like holliday endonuclease	Phi40_55

2732	RuvC-Like holliday endonuclease	Phi43_53
2733	RuvC-Like holliday endonuclease	Phi44_51
2734	RuvC-Like holliday endonuclease	Phi4_50
2735	RuvC-Like holliday endonuclease	Phi5.12_52
2736	RuvC-Like holliday endonuclease	Phi91127_55
2737	RuvC-Like holliday endonuclease	Phi93_54
2738	RuvC-Like holliday endonuclease	PhiA1127_51
2739	RuvC-Like holliday endonuclease	PhiA16_48
2740	RuvC-Like holliday endonuclease	PhiB1127_52
2741	RuvC-Like holliday endonuclease	PhiC0139_52
2742	RuvC-Like holliday endonuclease	PhiD.18_52
2743	RuvC-Like holliday endonuclease	PhiE1127_58
2744	RuvC-Like holliday endonuclease	PhiF.17_55
2745	RuvC-Like holliday endonuclease	PhiF0139_55
2746	RuvC-Like holliday endonuclease	PhiG_56
2747	RuvC-Like holliday endonuclease	PhiJF1_56
2748	RuvC-Like holliday endonuclease	PhiL.18_54
2749	RuvC-Like holliday endonuclease	PhiL.6_57
2750	RuvC-Like holliday endonuclease	PhiLj_48
2751	RuvC-Like holliday endonuclease	PhiM.16_53
2752	RuvC-Like holliday endonuclease	PhiM.5_56
2753	RuvC-Like holliday endonuclease	PhiM1127_58
2754	RuvC-Like holliday endonuclease	PhiS0139_52
2755	RuvC-Like holliday endonuclease	SL4_0050
2756	RuvC-Like holliday endonuclease	ViridisJM2_gp58
2757	RuvC-Like holliday endonuclease	bIBB29_gp53
2758	RuvC-Like holliday endonuclease	bIL170p63
2759	RuvC-Like holliday endonuclease	fd13_0052
2760	RuvC-Like holliday endonuclease	jj50_ORF48
2761	RuvC-Like holliday endonuclease	jm1_0050
2762	RuvC-Like holliday endonuclease	p2_0048
2763	RuvC-Like holliday endonuclease	phi7_55
2764	RuvC-Like holliday endonuclease	sk1p55
2765		
